# Au complexes with 5-S-alkylated-2-amino-1,3,4-thiadiazoles: from Au(III)-based ionic liquids to rare dinuclear Au(II) coordination compounds

**DOI:** 10.1038/s41598-026-57927-y

**Published:** 2026-06-18

**Authors:** Madalin Damoc, Alexandru-Constantin Stoica, Mihaela Silion, Ana-Maria Macsim, Aurica Farcas, Mihaela Dascalu, Sergiu Shova, Maria Cazacu

**Affiliations:** 1 Department of Inorganic Polymers, “Petru Poni” Institute of Macromolecular Chemistry, Aleea Gr. Ghica Voda 41A, Romanian Academy, 700487 Iasi, Romania; 2 Department of Physics of Polymers and Polymeric Materials, Petru Poni” Institute of Macromolecular ChemistryAleea Gr. Ghica Voda 41A, Romanian Academy, 700487 Iasi, Romania; 3 NMR Laboratory, Petru Poni” Institute of Macromolecular ChemistryAleea Gr. Ghica Voda 41A, Romanian Academy, 700487 Iasi, Romania; 4 Department of Electroactive Polymers and Plasmochemistry, Petru Poni” Institute of Macromolecular ChemistryAleea Gr. Ghica Voda 41A, Romanian Academy, 700487 Iasi, Romania

**Keywords:** Gold compounds, Ionic Au(III) complexes, Ionic liquid, Electrochemical behavior, Dinuclear Au(II) complexes, Flexible-tailed ligands, Chemistry, Materials science

## Abstract

The reaction of 5-amino-1,3,4-thiadiazole-2-thiols, S-modified with highly flexible trimethylsilylpropyl (H₂L^1^) and *n*-hexyl (H₂L^2^) groups, with HAuCl₄ afforded ionic salts of the type (H_3_L^n^)^(+)^[AuCl_4_]^(-)^ (n = 1, 2). The structures of the resulting complexes were elucidated by single-crystal X-ray crystallography (SC-XRD) and/or spectroscopic analyses, whose results support their ionic nature. Based on their appearance and DSC data, (H_3_L^1^)[AuCl_4_] melts at 55 °C, whereas (H_3_L^2^)[AuCl_4_] is liquid at room temperature. The electroactive behavior of both cations and anions in such environments, as demonstrated by cyclic voltammetry for (H_3_L^2^)[AuCl_4_], reflects their mobility and ability to participate in redox processes and is typical of ionic liquid electrolytes. Thus, these compounds show ionic liquid behavior at low temperatures. In the presence of water, rare dinuclear Au(II) complexes, [(HL^n^)_2_Au_2_Cl_2_], are formed, with a relatively short Au···Au distance, as demonstrated by crystallographic and additional analyses.

**Supplementary Information:**

The online version contains supplementary material available at https://doi.org/10.1038/s41598-026-57927-y.

## Introduction

Gold compounds play an important role in contemporary chemistry and materials science due to their ability to form stable complexes mainly in the +I and +III oxidation states with a wide variety of ligands, which gives them remarkable chemical, electronic, and biochemical properties^1–3^. In medicine and bioinorganic chemistry, these compounds are investigated as anticancer agents and as therapeutic alternatives to platinum-based drugs, acting on cellular proteins and enzymes through multiple mechanisms^1^.^,4–8^Beyond biomedical applications, gold compounds are used in the development of chemical sensors, luminescent materials, and supramolecular structures^9–11^, as well as as homogeneous catalysts^3^ or photocatalysts^12^in selective organic reactions and fine syntheses^13–16^. The growing interest in gold-catalyzed organic transformations is closely related to its unique electronic properties, driven by relativistic effects characteristic of heavy atoms. These effects lead to the contraction of the 6s orbitals and the relative stabilization of the 5d orbitals, significantly influencing the nature of chemical bonds and explaining the distinctive catalytic reactivity of gold in numerous organic transformations^13,14^.

In recent years, coordination gold chemistry has experienced significant development, especially due to advances in ligand design and understanding of reaction mechanisms, as highlighted in recent reviews in the field^17,18^. As mentioned above, the most stable and frequently encountered oxidation states of gold are +I and +III^19,20^, while Au(II) species are rare, thermodynamically unstable and tend to disproportionate in solution to Au(I) and Au(III)^19,21,22^. Au(II) exhibits Lewis acid behavior, combining the soft and hard acidity characteristics of Au(I) and Au(III), respectively^14^. In most cases, Au(II) complexes are observed as dinuclear species, stabilized by bridging ligands^23^and metal–metal interactions^20,23,24^, while mononuclear species are exceptional and difficult to isolate^14,20,24–29^. Stabilization of Au(II) species has, however, allowed the exploration of reactivity patterns comparable to those of Cu(II), providing valuable insights into the redox chemistry of gold^23,30^. A recent review highlights these aspects of Au(II) chemistry in detail, including experimentally confirmed structures and potential emerging applications^31^. Thus, Au(II) species have been proposed as transient reactive intermediates in homogeneous gold catalysis, particularly in redox and photoredox processes, and have also attracted increasing interest in biomedical research^14^. More recently, Au(II) complexes have been considered as potential key intermediates in artificial photosynthetic systems, where Au(III) fragments may act as electron acceptors. In addition, their involvement has been suggested in photoredox gold catalysis and in radical-type transformations associated with electron-transfer processes^31^

The ligand plays an essential role in gold chemistry, determining the oxidation state stability, geometry, and reactivity of the complexes^15^. For Au(I), the most widely used ligands are phosphines (R₃P) and N-heterocyclic carbenes (NHC), strong electron donors that stabilize the linear geometry and allow efficient applications in catalysis and organometallic synthesis^32^. Sulfur ligands, such as thiols and dithiocarbamates, exhibit high affinity for “soft” metal centers and can coordinate both Au(I) and Au(III). Au(III) complexes with dithiocarbamate ligands have demonstrated significant biological activity, including antiproliferative effects on cancer cells^8^. In the case of Au(III), polydentate ligands, such as nitrogen derivatives or macroazacycles, stabilize the square-planar geometry and allow fine control of electronic properties, being investigated in both catalysis and biological applications^16^. Thus, ligand selection allows for precise tuning of electronic and steric properties, directly influencing applications in catalysis, medicine, and functional materials.

Organic gold salts, such as tetrachloroaurate(III) of organic cations, are stable and soluble species in polar media, used as precursors for the synthesis of Au(I/III) complexes with specific ligands^33^and can exhibit ionic liquid behavior. These systems are of interest due to the redox potential of gold, relativistic effects and conductive properties, as well as due to the typical characteristics of ionic liquids, such as low volatility and high thermal stability^34,35^. The first systems of this type were reported by Hasan et al., including tetrachloroaurate salts of N,N-dialkylimidazolium, with melting points around 50 °C^36^. Subsequently, it was observed that most of these systems contain the AuCl₄⁻ anion associated with heterocyclic cations^37,38^. The ionic liquid behavior is mainly determined by weak intermolecular interactions between ions, rather than the flexibility of the alkyl chains, although steric effects can influence the transition to the liquid state at room temperature^39,40^.

In our recent work, we have developed ligands with flexible and hydrophobic segments for the synthesis of metal complexes of transition metals^41–44^and lanthanides^45^. Continuing these studies, in this work we used two 5-amino-2-mercapto-1,3,4-thiadiazole derivatives, S-alkylated with 3-(trimethylsilyl)propyl and *n*-hexyl groups, which were reacted with HAuCl₄ as a source of gold ions. The resulting ionic salts with organic cations exhibit ionic liquid behavior at or near room temperature, supported by spectral, thermal, and electrochemical measurements (cyclic voltammetry). An important aspect highlighted in this study is the fact that, in the presence of water, these compounds undergo structural transformations leading to the formation of dinuclear Au(II) complexes, the structures of which were confirmed by crystallographic analysis and complementary techniques (FTIR and NMR spectroscopy, elemental analysis, and MALDI-MS).

## Results and discussions

### Synthesis and structural characterization of the complexes

**Scheme 1:**
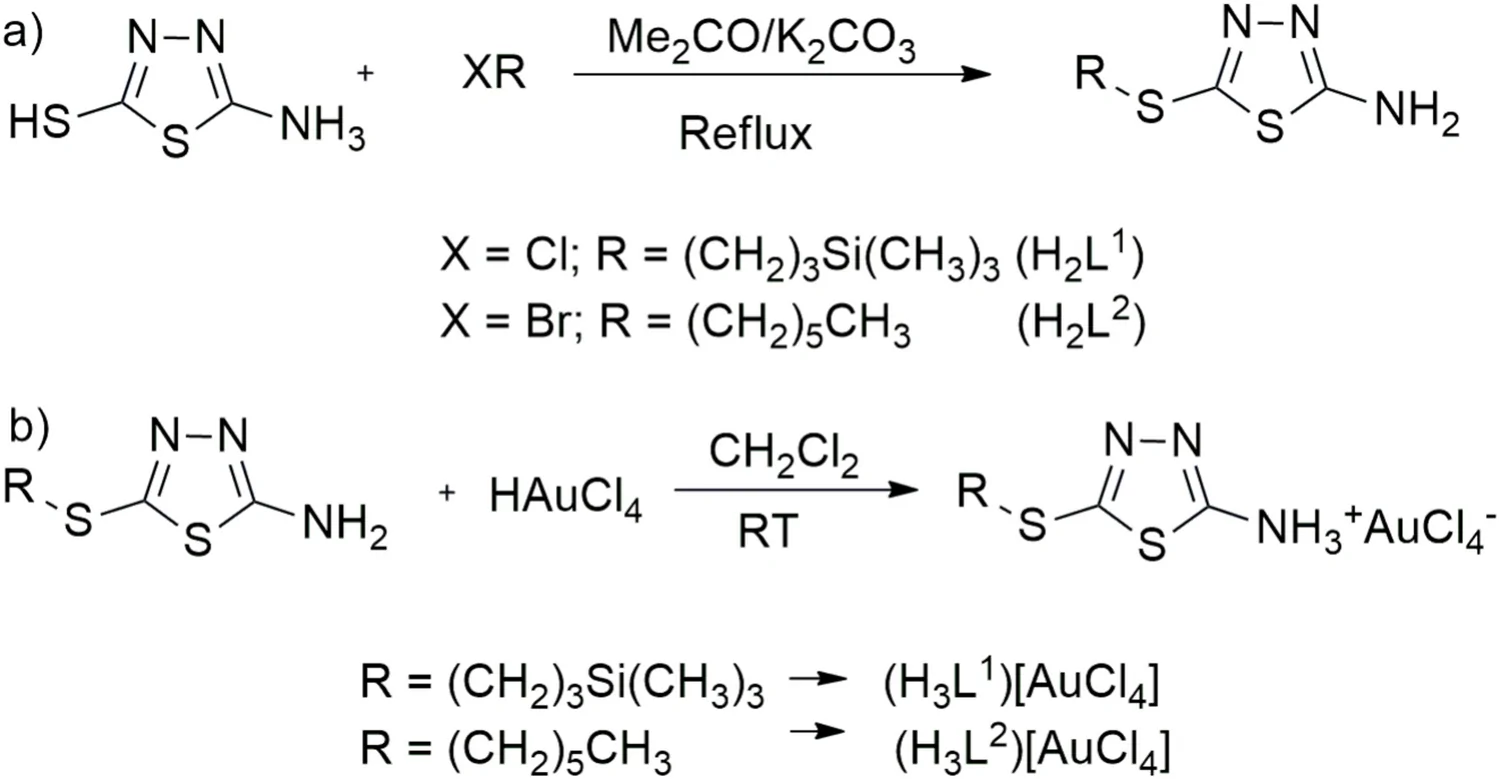
Reactions leading to the H_2_L^1 43^ and H_2_L^2^ ligands and their gold complexes.

**Fig. 1:**
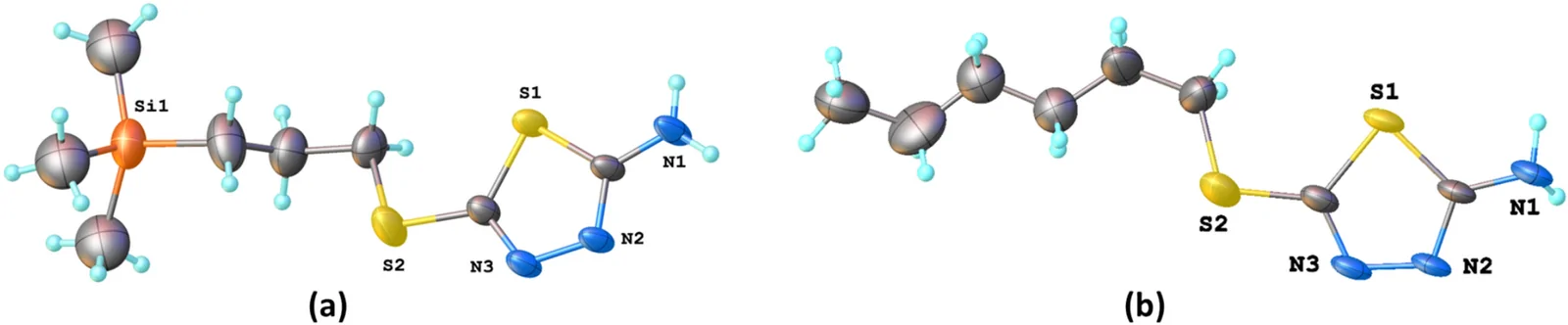
SC-XRD structure of H_2_L^1 43^ (**a**) and H_2_L^2^ (**b**), with thermal ellipsoids at 50% probability level.

Two compounds derived from 5-mercapto-2-amino-1,3,4-thiadiazole (Scheme 1a) were investigated as potential ligands for gold (Scheme 1b). One of them, S-alkylated with a trimethylsilylpropyl group (H_2_L^1^) (Fig. 1a), was previously reported by us^46^, while the second, bearing an *n*-hexyl substituent (H₂L^2^), was synthesized in this work (Scheme 1a).

The new ligand H_2_L^2^ was obtained in high yield (96.2%) via S-alkylation of 5-mercapto-2-amino-1,3,4-thiadiazole with 1-bromohexane in acetone, in the presence of K₂CO₃. Its structure was established by single-crystal X-ray diffraction analysis (Fig. 1b) of crystals grown from DMSO and further confirmed by IR, NMR, and elemental analysis of the isolated product (Figures S1–S3). The main crystallographic data and structure refinement parameters are summarized in Table S1, while selected interatomic distances, bond angles, and key hydrogen-bonding parameters are listed in Table S2.

**Fig. 2:**
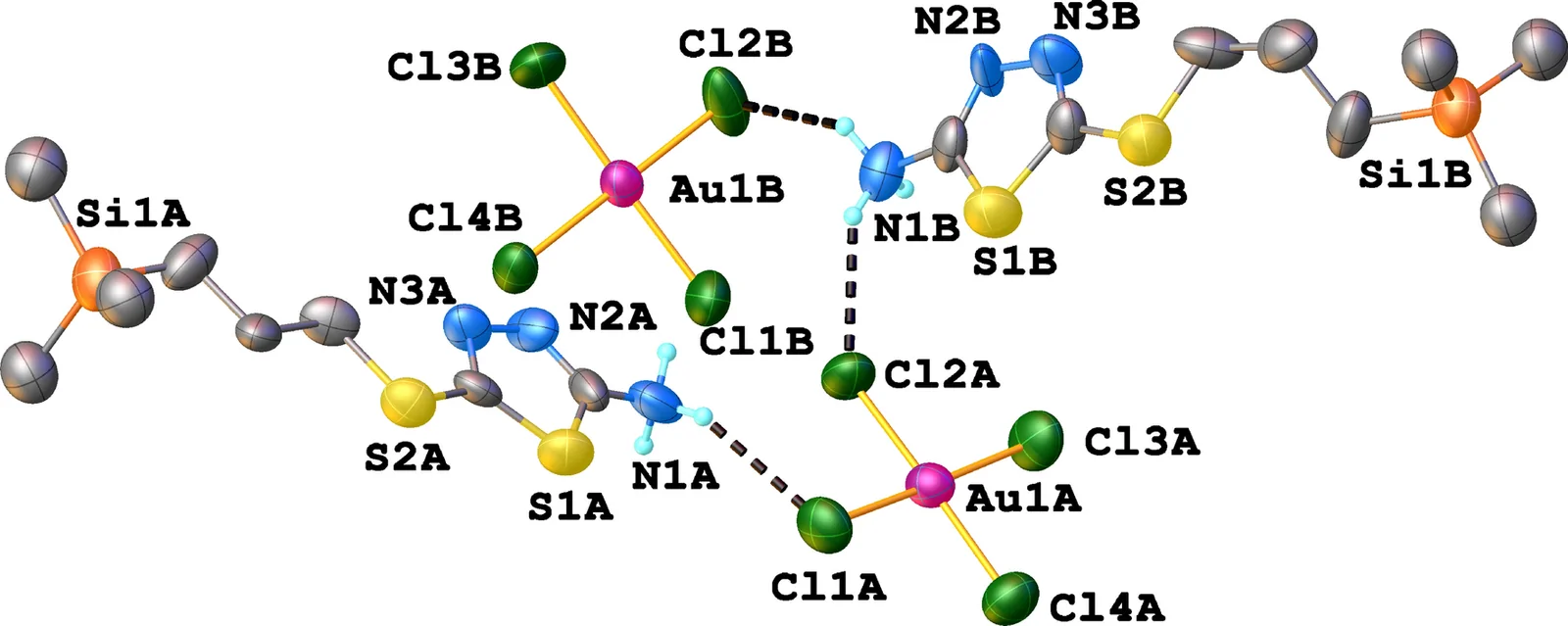
SC-XRD structure of (H_3_L^1^)[AuCl_4_] with thermal ellipsoids at 50% probability level. Irrelevant hydrogen atoms were removed for clarity; black dashed lines represent hydrogen bonds.

The H_2_L^1^ and H_2_L^2^ compounds were reacted with HAuCl_4_, in a 1:1.3 ratio, determined empirically through successive trials to improve reaction efficiency, under an inert atmosphere using standard Schlenk techniques (N₂). In both cases, the amino group becomes protonated, leading to ionic compounds of the general formula (H_3_L^n^)[AuCl_4_] (n = 1, 2), with [AuCl₄]⁻ as the counterion (Scheme 1b). The reaction products were purified by simple precipitation with *n*-hexane, followed by filtration, resulting in a yellowish solid (n = 1) or a yellowish liquid (n = 2) at room temperature. After isolation, the compounds were stored under an inert atmosphere. Crystals suitable for single crystal X-ray diffraction analysis were obtained only for (H_3_L^1^)[AuCl_4_], either directly from the reaction mixture or from diluted CH_2_Cl_2_/Et_2_O solutions. X-ray analysis of (H_3_L^1^)[AuCl_4_] (Fig. 2, Tables S3–S4) revealed that the asymmetric unit contains two ammonium cations and two independent AuCl₄⁻ counterions, labeled A and B.

Since the (H_3_L^2^)[AuCl_4_] compounds could not be crystallized adequately for SC-XRD analysis, its structure was confirmed by other methods, as discussed below.

IR and NMR data (Figures S4-S9) for gold complexes are consistent with the structures determined by crystallography (Figure 2) or those proposed in Scheme 1.  As a result of complexation, the IR absorption bands corresponding to the N–H stretching and –NH₂ scissoring vibrations of the primary amino groups in the ligands shift to higher wavenumbers: from 3253 and 1616 cm^−1^ in H₂L^1^ to 3395 and 1622 cm^−1^ in (H_3_L^1^)[AuCl_4_], and from 3277 and 1595 cm^−1^ in H_2_L^2^ to 3389 and 1624 cm^−1^ in (H_3_L^2^)[AuCl_4_]. This blue shift can be attributed primarily to the intramolecular stiffening of the N–H bonds upon protonation^47^. The bands at 498 cm^−1^ (Figure S4) and 482 cm^−1 ^(Figure S5) in the IR spectra of the compounds (H₃Lⁿ)[AuCl₄] (n = 1, 2) can be attributed to Au–Cl stretching vibrations^40^. In ^1^H NMR, the NH_3_ protons of (H_3_L^1^)[AuCl_4_] (Figure S6) and (H_3_L^2^)[AuCl_4_] (Figure S8) appear at 8.08 and 7.80 ppm, respectively^48,49^, downshifted as compared to 7.27 and 7.28 ppm in the case of pure H_2_L^1^ and H_2_L^2^ ligands, supporting ionic nature of the complexes. In ^13^C{^1^H} NMR spectra, the carbon atoms in the thiadiazole ring also exhibit shifts in the complex relative to the ligand, as can be observed in Figures S7, S10 and from the values presented in Table 1.

**Table 1:** Changes in the chemical shifts of the ^1^H and ^13^C NMR spectra for the (H_3_L^n^)[AuCl_4_] complexes compared to the corresponding free ligands.


**Group**	**H**_**2**_**L**^**1**^	**(H**_**3**_**L**^**1**^**)[AuCl**_**4**_**]**	**Δδ**	**H**_**2**_**L**^**3**^	**(H**_**3**_**L**^**2**^**)[AuCl**_**4**_**]**	**Δδ**
^1^H NMR, δ (ppm)						
NH₂/NH_3_^+^	7.27	8.08	0.81	7.28	7.80	0.52
^13^C NMR, δ (ppm)						
C2	150.18	152.98	2.80	150.23	151.53	1.30
C5	169.36	169.59	0.23	169.39	169.97	0.58

Thus, the signal of C2, linked to the S-alkyl group, is shifted by 2.8 and 1.3 ppm, while the signal of C5, bearing the NH_2_ group, is shifted by 0.23 and 0.58 ppm in the complexes (H_3_L^1^)[AuCl_4_] and (H_3_L^2^)[AuCl_4_], respectively, as compared with the corresponding ligands. In MALDI-MS spectra, signals corresponding to gold compounds were observed at *m/z* 584.924 for (H_3_L^1^)[AuCl_4_] (theoretical: 584.913) and 554.930 for (H_3_L^2^)[AuCl_4_] (theoretical: 554.920).

### Ionic liquid behavior

**Fig. 3:**
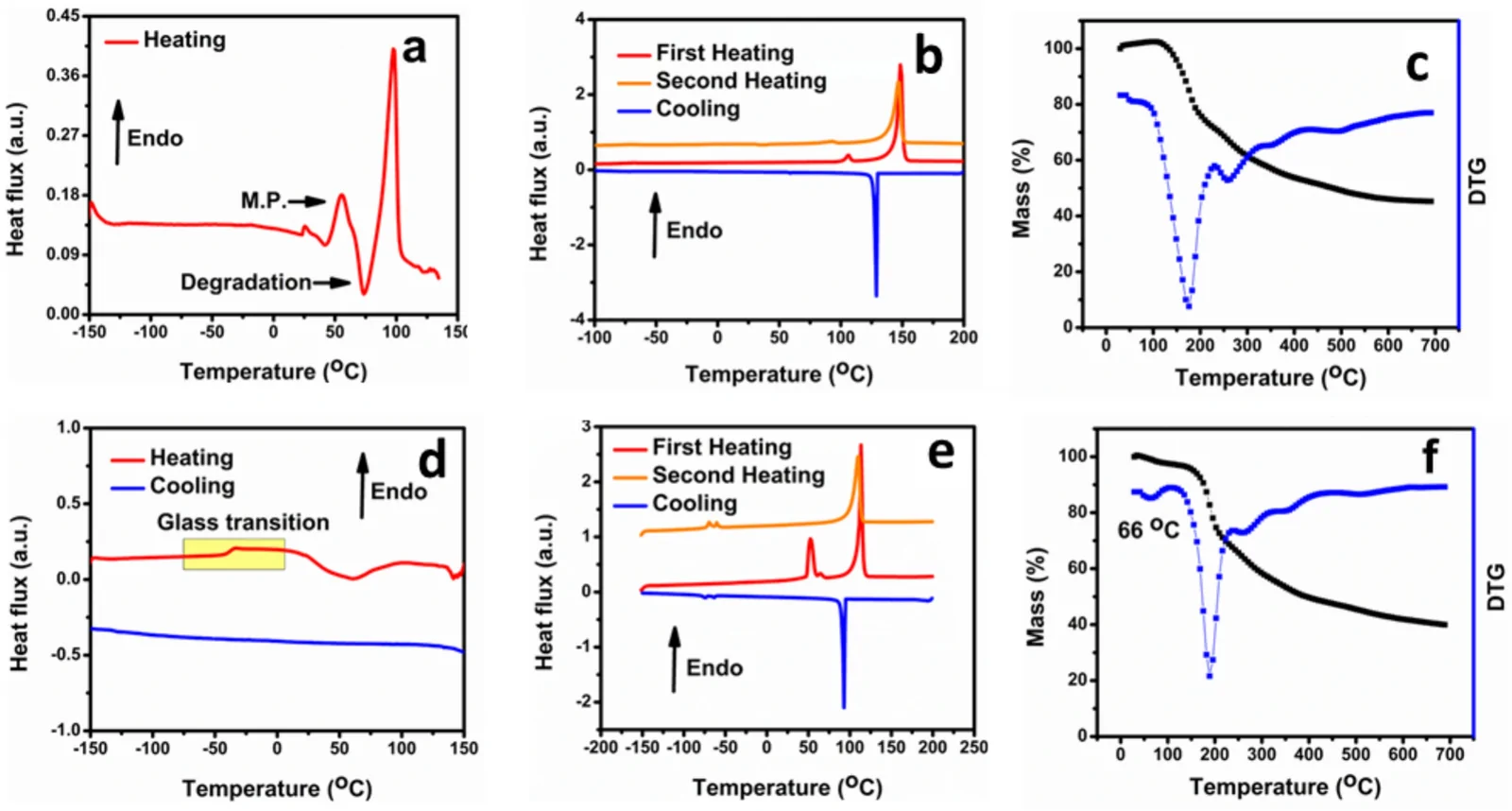
DSC curves: (H_3_L^1^)[AuCl_4_] (**a**); H_2_L^1^ (**b**); (H_3_L^2^)[AuCl_4_] (**d**); H_2_L^2^ (**e**). TGA curves: (H_3_L^1^)[AuCl_4_] (**c**) and (H_3_L^2^)[AuCl_4_] (**f**).

The oily state of the (H_3_L^2^)[AuCl_4_] at room temperature and the fact that, in the case of the (H_3_L^1^)[AuCl_4_] analogue, prepared under similar conditions but isolated in crystalline form, SC-XRD analysis revealed the formation of an ionic complex, suggest that such compounds may exhibit ionic liquid behavior at low temperatures. DSC analysis shows that (H_3_L^1^)[AuCl_4_] displays a melting point of 55 °C (Fig. 3a), while (H_3_L^2^)[AuCl_4_] exhibits a glass transition, Tg, at –37 °C (Fig. 3d), as reported for other amorphous ionic systems and related low-melting ionic materials^50–52^. These transitions are lower than those of the corresponding ligands, namely 149 °C for H_2_L^1^ and 114 °C for H_2_L^2^ (Figs. 2b,2e). This decrease is quite interesting and is likely due to reduced lattice energy arising from weak ionic interactions and their conformational flexibility, further enhanced by the flexible alkyl and silicon-containing moieties. For example, in the crystal structure of (H_3_L^1^)[AuCl_4_], the shortest interactions between the two ions are N–H···Cl of 2.63–2.80 Å (H···Cl distance), indicating relatively weak interactions that contribute to the compound’s structural flexibility. A gold compound with ionic liquid behaviour above −37 ºC is notable and comparable with that of widely spread organic ionic liquids. However, their thermal stability, determined by thermogravimetric analysis (TGA), is relatively low, i.e., 73 ºC for (H_3_L^1^)[AuCl_4_] and 66 ºC for (H_3_L^2^)[AuCl_4_] (Fig. 3c, 3f), lower than that of the corresponding ligands (Figure S10).

To verify that their phase transition is not due to liquid crystal behavior, both complexes were examined using polarized optical microscopy (POM). At the aforementioned transition temperatures, they showed no liquid crystalline textures but only isotropic features^53^. Future work will focus on stabilizing the coordination environment of gold to improve thermal and long-term operational stability for potential applications.

### Redox behavior of (H_3_L^2^)[AuCl_4_]

**Fig. 4:**
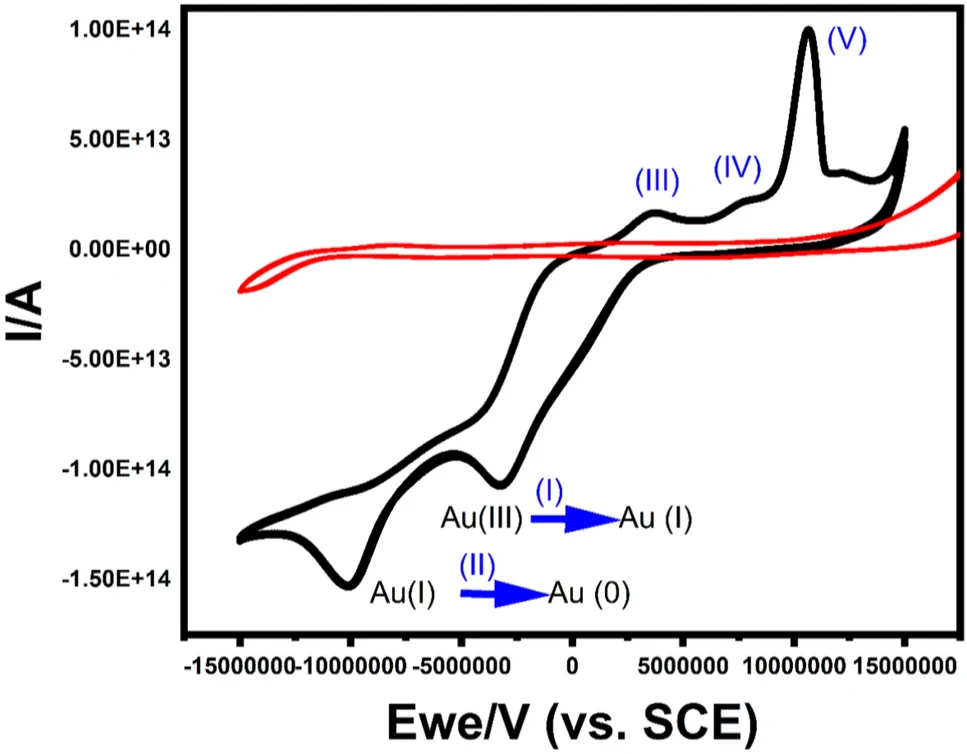
Cyclic voltammograms of the supporting electrolyte (red curve) and 1.3 mM (H_3_L^2^)[AuCl_4_] (black curve) in acetonitrile containing 0.1 M [NBu_4_][PF_6_], recorded at scan rate of 50 mV/s.

To gain further insight into the electrochemical properties of the ionic compounds, cyclic voltammetry (CV) was employed. To better understand the factors governing the ionic liquid-like behavior at room temperature, more interesting compound, (H_3_L^2^)[AuCl_4_], which is liquid at room temperature, was selected for detailed electrochemical investigation. Since tetrabutylammonium hexafluorophosphate, [NBu_4_][PF_6_], as the supporting electrolyte shows no voltammetric peak within the experimental potential range, all observed redox peaks in the CVs arises from the [AuCl₄]^(^⁻^)^/(H₃L^2^)^(^⁺^)^ ionic compound (Fig. 4). The cathodic peaks at −0.03 V (I) and −0.80 V (II) vs SCE correspond to the two-step reductions of gold: from Au(III) to Au(I) and from Au(I) to Au(0)^37^. In the anodic region, oxidation peaks at 0.67 V (III) and 1.09 V (IV) could be attributed to chloride oxidation to chlorine, according to the literature^37^. In complex electrochemical environments, this process can proceed through multiple, surface-dependent steps involving adsorption and secondary redox processes, which may account for the observed multiple anodic signals^40^. The third peak at 1.35 V (V) likely arises from the oxidative dissolution, or anodic stripping, of Au(0) deposited on the platinum electrode surface, converting it back to Au(I)^37^. The electroactive behavior of both cations and anions in such environments, reflecting their mobility and ability to participate in redox processes, is typical of ionic liquid electrolytes, as has been well documented in the literature^54^. Fig. 4 shows the cyclic voltammogram of the supporting electrolyte and a 1.3 mM sample dissolved in the same electrolyte.

### Effect of water on ionic gold species

**Scheme 2:**
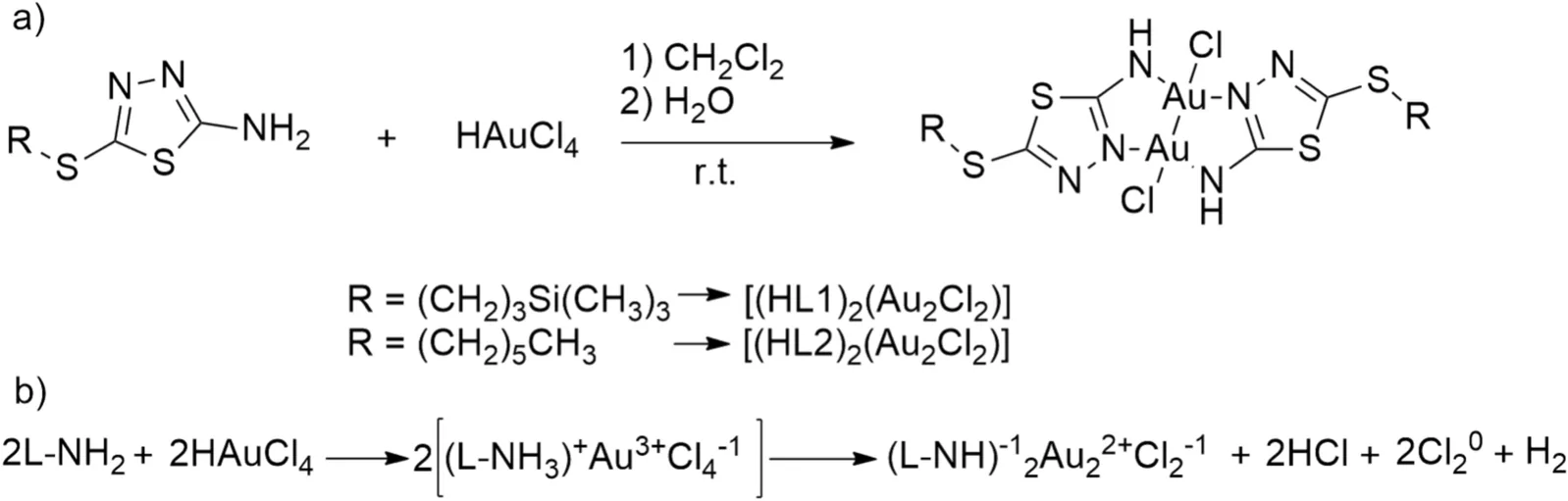
Formation of Au(II) complexes (**a**) and the redox process assumed to underlie it (**b**).

To investigate the influence of water on the formation of the target Au(III) complexes^55^, 1:1 reactions of the ligands with HAuCl₄ in methylene chloride were carried out, followed by the deliberate addition of water. This approach was used to evaluate changes in the reaction outcome in the presence of water. The reaction proceeds in two steps: first, HAuCl₄ and H₂L^n^ (n = 1, 2) are reacted under inert conditions in CH₂Cl₂ to generate the ionic gold(III) species; then water is added, and the mixture is stirred overnight. This procedure led to the formation of dinuclear Au(II) complexes rather than the original ionic gold species (Scheme 2a), as revealed by the structural analysis presented below.

Exposure of (H_3_L^n^)[AuCl_4_] to water leads to significant changes in the coordination environment of the Au(III) center. It is well established that tetrachloroaurate(III) undergoes hydrolysis in aqueous media, involving stepwise substitution of chloride ligands by aqua and/or hydroxo groups, thereby altering the electronic and structural properties of the complex^56^. Such ligand exchange processes are well documented for [AuCl₄]⁻^57^and are known to affect the redox properties of Au(III) complexes in solution, and Au(III) species are generally considered redox-active and susceptible to reduction under appropriate conditions^58^. Moreover, dinuclear Au(II) complexes featuring Au–Au interactions and bridging chloride ligands have been structurally characterized, demonstrating that Au(II) can be stabilized in dimeric motifs of the general type [Au₂Cl₂L₂]^59^.

The separation of the dinuclear Au(II) compounds was achieved by precipitation with *n*-hexane; however, simple filtration did not yield pure compounds. Both Au(II) complexes were purified by repeated centrifugation after precipitation. The compounds were obtained as purple solids in moderate yields, up to 50%.

**Fig. 5:**
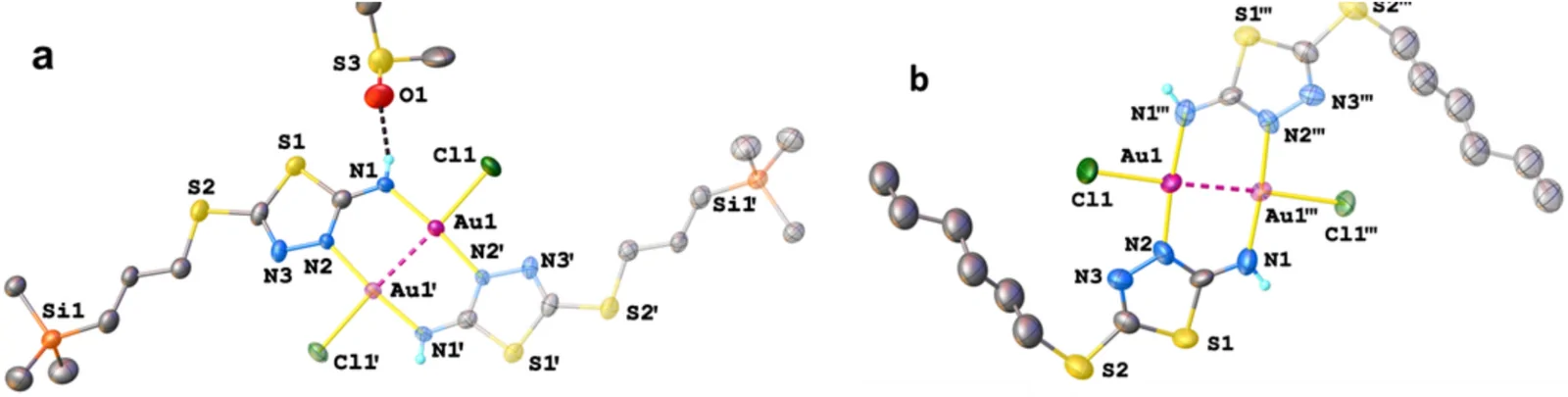
SC-XRD structures of Au(II) complexes: [(HL^1^)_2_Au_2_Cl_2_] (**a**) and [(HL^2^)_2_Au_2_Cl_2_] (**b**), with thermal ellipsoids at a 50% probability level. Irrelevant hydrogen atoms were removed for clarity; black dashed lines represent hydrogen bonds. Symmetry codes: ^')^ 2 – *x*, 1 – *y*, 2 – *z*; ^''')^ 1 – *x*, 1 – *y*, 1 – *z*.

The crystals for X-ray diffraction of [(HL^1^)_2_Au_2_Cl_2_] and [(HL^2^)_2_Au_2_Cl_2_] were grown from DMSO solutions. According to crystallographic analysis data (Tables S5–S7), both compounds adopt a similar dinuclear structure, as shown in Fig. 5. Each Au^2+^ ion is coordinated by two bidentate organic ligands and one chloride anion, in a slightly distorted square planar geometry. The Au···Au′ contacts are observed at 2.5383(5) Å in [(HL^1^)_2_Au_2_Cl_2_] and 2.5299(18) Å in [(HL^2^)_2_Au_2_Cl_2_], generally somewhat shorter but close to those reported in the literature for dinuclear Au(II) compounds with different ligands^19,21,24–28^. These include, for example, values of 2.5454 Å, 2.5898(6) Å and 2.5960(7) Å, as well as 2.679(1) Å, in dinuclear Au(II) complexes containing different classes of ligands, namely (CF₂)ₙ bridging chains^19^, PPh₂–aromatic^26^, and (CF_3_)_2_(py)^60^ ligands. These values arise from the rigidity of the complexing group based on 5-amino-1,3,4-thiadiazole-2-thiol, which exhibits extended conjugation. This structural feature enforces the positioning of the two gold centers, leading to the formation of N–Au–N′ angles of 174° and 175° in the two compounds, respectively, while the Cl–Au···Au–Cl unit is also almost linear (Tables S6, S7). This geometry is not significantly influenced by intermolecular forces, due to the specific nature of the ligands employed, which possess flexible, nonpolar tails (trimethylsilyl ethyl or hexyl). Au-Au bonding increases stability, and Au₂^4+^core derivatives represent the most stable and common Au(II) complexes^23^. From another point of view, the synthesis of the dinuclear Au(II) complexes can be seen as a double redox process involving the reduction of the Au^3+^ metal center to Au^2+^ and the -NH_3_^+^ cations to a secondary amine -NH-. A presumable redox process is depicted in Scheme 2b. Based on this assumption the Cl^−1^ is oxidized to Cl^0^.

**Fig. 6:**
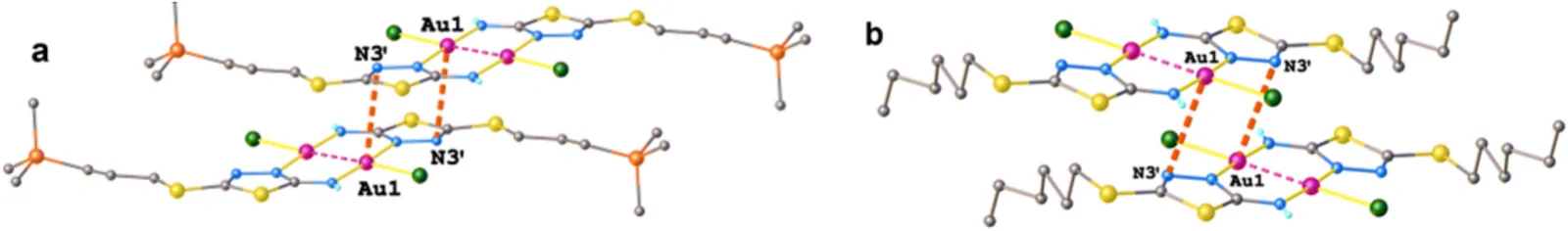
[4 + 1] coordination geometry of Au(II) complexes: [(HL^1^)_2_Au_2_Cl_2_] (**a**), (HL^2^)_2_(Au_2_Cl_2_) (**b**).

A detailed analysis of the crystal structures for each Au(II) complex revealed, besides the intramolecular bonding, an additional intermolecular contact of Au^2+^ ions to N atoms of adjacent molecules in apical positions: Au···N' of 3.493(6) Å for [(HL^1^)_2_Au_2_Cl_2_], and Au···N' of 3.563(19) Å for [(HL^2^)_2_Au_2_Cl_2_] (Fig. 6). Due to the additional intermolecular bonding, which is significantly longer, the coordination geometry can be considered as a [4 + 1] square pyramid^61^.

In the FTIR spectra of the two complexes, [(HL^1^)_2_Au_2_Cl_2_] and (HL^2^)_2_(Au_2_Cl_2_), bands corresponding to N–H stretching and –NH₂ scissoring vibrations are observed at 3277 and 1614 cm^−1^, and 3296 and 1622 cm^−1^, respectively (Figures S11, S12), while those assigned to the C–N vibrations of the thiadiazole ring appear at 1535 and 1539 cm^−1^. In the case of the complex (H₂L₁)₂(Au₂Cl₂), a band at 1246 cm^−1 ^is present, which is characteristic of the Si–CH₃ group. The Au–Cl band is not visible within the recorded FTIR spectral range, likely being located in the far-IR region^62^.

^1^H NMR spectra of the [(HL^1^)_2_Au_2_Cl_2_] and [(HL^2^)_2_Au_2_Cl_2_] compounds (Figures S13, S14) show the signal for the -NH proton at 8.24 and 8.25 ppm, downshifted as compared with pure ligands (7.27 and 7.28 ppm, respectively). ^13^C NMR analysis could not be performed because all complexes degrade overnight in solution, regardless of the solvent, most likely by disproportionation to Au(I) and Au(III), as previously reported ^21^. Their purity was further verified through elemental analysis, as showed in the Experimental section.

**Fig. 7:**
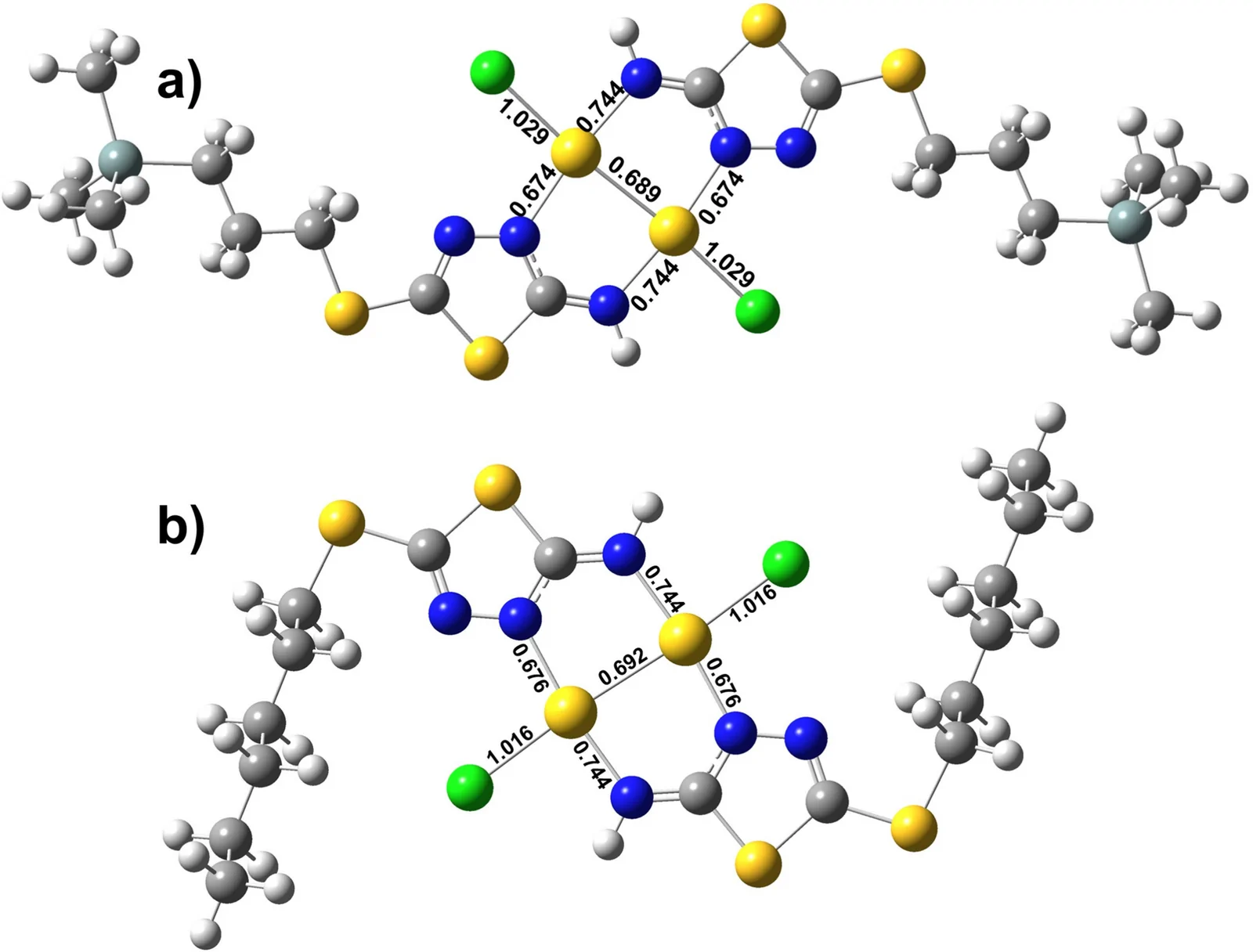
Selected Wiberg bond order for: (**a**) [(HL^1^)_2_Au_2_Cl_2_]; (**b**) [(HL^2^)_2_Au_2_Cl_2_].

The [(HL^1^)_2_Au_2_Cl_2_] and [(HL^2^)_2_Au_2_Cl_2_] structures were optimized using Gaussian 16 software, with the B3LYP method and the DEF2SVP basis set. The optimization process was validated by frequency calculation, yielding no negative values. The XYZ matrix for the optimized molecules is shown in Tables S8-S9. NBO analysis was also performed, on the basis of which the Wiberg bond order was calculated (Tables S10–S11). The calculated Wiberg order for the [(HL^1^)_2_Au_2_Cl_2_] molecule was 0.689796 for the Au···Au interaction, which is considerably less than 1 but still cannot be neglected (Fig. 7a). For the [(HL^2^)_2_Au_2_Cl_2_] molecule, a bond order of 0.692009 was calculated, which is also significantly lower 1 (Fig. 7b). However, the values ​​cannot be neglected, indicating a specific aurophilic interaction^63^.

## Conclusions

The ligands based on 5-amino-1,3,4-thiadiazole-2-thiol S-modified with flexible trimethylsilylpropyl (H₂L1) and *n*-hexyl tails (H_2_L^1^, H_2_L^2^) form stable ionic complexes with HAuCl₄ of the type (H_3_L^n^)[AuCl_4_] (n = 1,2). SC-XRD analysis of compound (H_3_L^1^)[AuCl_4_] confirmed its ionic nature, while NMR analysis of both compounds showed downfield shifts of the NH protons, indicating strong cation–anion interactions. (H_3_L^2^)[AuCl_4_] is liquid at room temperature, and (H_3_L^1^)[AuCl_4_] melts at 55 °C, indicating that these complexes may show behavior consistent with ionic liquids at low temperatures. Cyclic voltammetry further confirmed the mobility and electroactivity of the [AuCl₄]⁻ anion, consistent with the behavior expected for protic ionic systems. Moreover, in the presence of water, these complexes undergo tnansformation into rare dinuclear Au(II) species, [(HL^n^)_2_Au_2_Cl_2_], where the two bidentate thiadiazole-based ligands bridges the two Au^2^⁺ centers, and each Au atom is additionaly coordinated by one chloride anion, in a slightly distorted square-planar geometry. The N–Au–N′ angles and the Cl–Au–Au–Cl sequences are close to linearity, while the Au···Au′ distance is 2.5383(5) Å for n = 1 and 2.5293(18) Å in for n = 2, slightly shorter than those reported in the literature for complexes involving other ligands, indicating a greater stabilization of the dinuclear complex. Overall, these findings highlight the potential of (H_3_L^2^)[AuCl_4_] complexes as tunable ionic liquid-like materials with electrochemical activity and structural versatility.

## Experimental

### Materials

5-amino-2-S-alkylated-1,3,4-thiadiazoles (only H_2_L^1^ and H_2_L^2^) were synthesised according to previous publications^1^. HAuCl_4_ x H_2_O (99.99%), 1-bromohexane (98%), methylene chloride, n-hexane, acetone, diethylether, and dimethylsulfoxide were purchased from Sigma-Aldrich. Before using the solvents, they were dried over molecular sieves and flushed with nitrogen. Potassium carbonate anhydrous (99%) was purchased from Fluka and dried in the oven before using it. HAuCl_4_ was dried before using it and stored in an inert atmosphere.

### Measurements

FTIR (Fourier transform infrared) spectra were recorded using a Bruker Vertex 70 FT-IR spectrometer. The measurements were performed in the transmission mode in the range 400–4000 cm^−1^ at room temperature with a resolution of 2 cm^−1^ and an accumulation of 32 scans. NMR (Nuclear magnetic resonance) spectra were recorded on a Bruker Advance NEO 400 MHz Spectrometer equipped with a 5 mm QNP direct detection probe and z-gradients. The spectra were recorded in DMSO-d_6_ at room temperature, and the chemical shifts are reported as δ values (ppm) using the solvent residual peak (2.51 for ^1^H and 39.5 for ^13^C) as reference. Elemental Analysis: Carbon, hydrogen, nitrogen, and sulfur content were determined on a Perkin–Elmer CHNS 2400 II elemental analyser. MALDI-MS (Matrix-assisted laser desorption/ionisation mass spectrometry): The presented MS spectra were acquired using Bruker’s RapifleX MALDI ToF/ToF. The spectra were processed using FlexAnalysis 4.0 software (Bruker Daltonics, Bremen-Germany). The samples (1 mg/mL) and the matrix (α-cyano-4-hydroxycinnamic acid—CHCA) (20 mg/mL) were prepared in acetonitrile. The thin layer technique was used for sample deposition on the ground steel target. SC-XRD (Single-crystal X-ray diffraction): Crystallographic measurements were carried out with a Rigaku Oxford-Diffraction XCALIBUR E CCD diffractometer equipped with MoKα radiation. The unit cell determination and data integration were carried out by using the CrysAlis package of Oxford Diffraction^64^. The structures were solved by Intrinsic Phasing using Olex2 software with the SHELXT structure solution program and refined by full-matrix least-squares on *F*^*2*^with SHELXL-2015 using the anisotropic model for non-hydrogen atoms^65,66^. All H atoms bonded to carbon were introduced in idealised positions using the riding model. The positions of the hydrogen atoms in the NH groups were verified using the electron density map. They were also evaluated in terms of the hydrogen bonds they form. The hydrogen bond parameters are provided in the Supporting Information. CCDC-2390298 (H_2_L^3^), CCDC-2390300 ([H_3_L^1^][AuCl_4_]), CCDC-2257489 ([(HL^1^)_2_Au_2_Cl_2_]), CCDC-2257490 ([(HL^2^)_2_Au_2_Cl_2_]), contain the supplementary crystallographic data for this contribution. Whenever necessary, restraints were imposed on geometry and displacement parameters of disordered molecules. For (H_3_L^1^)[AuCl_4_], which crystallizes in the *Pca*2_1_ space group, an inversion twinning was applied with the refined BASF parameter of 0.40(4). For [(HL^2^)_2_(Au_2_Cl_2_)], which crystallizes in the *P*−1 space group, a twinning was applied (law 1.0001 0.0007 0.0021 −0.0108 0.9953 −0.0167 0.0181 0.0743 1.0018) with the refined BASF parameter of 0.494(9). These data can be obtained free of charge via www.ccdc.cam.ac.uk/conts/retrieving.html (or from the Cambridge Crystallographic Data Centre, 12 Union Road, Cambridge CB2 1EZ, UK; fax: (+ 44) 1223–336-033; or deposit@ccdc.ca.ac.uk). TGA (Thermogravimetric analysis) was performed on STA 449F1 Jupiter NETZSCH (Germany) equipment. The measurements were made in the temperature range of 20–700 °C under a nitrogen flow (50 mL/min) using a heating rate of 10 °C/min. An alumina crucible was used as a sample holder. DSC (Differential scanning calorimetry) measurements were performed on a DSC 200 F3 Maia (Netzsch, Germany) in the range −150 ÷ 200 °C, applying a heating and cooling rate of 5 °C/min. About 10 mg of sample were heated in pressed and punched aluminium crucibles using nitrogen as a flowing inert purge gas at a flow rate of 100 mL/min. POM (Polarised optical microscopy): Images were recorded with a Zeiss Axio Imager.A2m, camera Axiocam 208 cc, Carl Zeiss AG, Oberkochen, Germany, with a heating and cooling rate of 5 °C/min. CV (Cyclic Voltametry): Electrochemical measurements were performed on a biologic VSP workstation in acetonitrile and tetrabutylammonium hexafluorophosphate, [NBu_4_][PF_6_], as the supporting electrolyte. Cyclic voltammetry traces were performed at room temperature with a conventional three-electrode cell configuration, a 1 mm Pt radius electrode, a platinum wire and a saturated calomel electrode (SCE) were used as the working, counter and reference electrodes, respectively.

### Procedure

#### H_2_L^2^ synthesis

1 g (7.50 mmol) of 5-amino-2-mercapto-1,3,4-thiadiazole were dissolved in 50 mL acetone, then 1.0375 g (7.50 mmol) of K_2_CO_3_ were added at 65 °C. In time, a precipitate appeared and the mixture color turned in light blue. After two hours, 810 μL (5.77 mmol) of 1-bromohexane were added and the mixture colour turned white. The mixture was kept under reflux for two days. Afterwards, the white precipitate was filtered and several times washed with water, acetone, and petroleum ether. The product was crystallised from DMSO as white squares. Yield 1.2064 g (96.2%). IR (KBr), cm^−1^: ν_max_ 3277 (NH_2_) and 1595 (NH_2_), 1514vs (thiadiazole), 1425 s (-CH_2_-). ^1^H NMR (DMSO-*d*_*6*_, 400.13 MHz): δ (ppm) 7.28 (s, 2H, NH_2_), 3.04 (t, *J* = *7.27 Hz*, 2H, S-CH_2_), 1.62 (quintet, J = 7.23 Hz, 2H, CH_2_), 1.37 (quintet, J = 7.37 Hz, 2H, CH_2_), 1.30 − 1.24 (m, 4H, CH_2_), 0.87 (t, *J* = *6.92 Hz*, 3H, CH_3_). ^13^C RMN (DMSO-*d*_*6*_, 100.62 MHz): δ (ppm) 169.39 (C-N), 150.23 (C-S), 34.30 (CH_2_-S), 30.63 (CH_2_), 28.99 (CH_2_), 27.46 (CH_2_), 21.93 (CH_2_), 13.82 (CH_3_). Anal. Calcd (%) for C_8_H_15_N_3_S_2_ (Mw 217.35 g mol^−1^): C, 44.21; H, 6.96; N, 19.33; S, 29.50. Found: C, 44.01; H, 6.96; N, 19.33; S, 29.79.

#### (H_3_L^1^)[AuCl_4_] synthesis

77.4 mg (0.18 mmol) of HAuCl_4_ and 35.7 mg (0.14 mmol) of H_2_L^1^ were dissolved in 10 mL of CH_2_Cl_2_ under Schlenk conditions (N_2_) and mixed at room temperature overnight. Afterwards, 30 mL of n-hexane were added, resulting a yellowish precipitate, which was collected by filtration and washed with fresh n-hexane. Crystals were grown from CH_2_Cl_2_/Et_2_O by solvents’ slow evaporation. Yield: 42.8 mg (50.0%). IR (KBr), cm^−1^: ν_max_ 3395, 3304, 3223, 3169 (-NH_3_), 1622 (-NH_3_), 1568 (thiadiazole), 1246, 864, 835 (Si-CH_3_). ^1^H NMR (DMSO-*d*_*6*_, 400.13 MHz): δ (ppm) 8.08 (s, 2H, -NH_3_), 3.10 (t, *J* = *7.27 Hz*, 2H, S-CH_2_), 1.69 − 1.61 (m, 2H, -CH_2_-), 0.61 (quintet, J = 3.60 Hz, 2H, Si-CH_2_), −0.01 (s, 9H, Si-CH_3_). ^13^C NMR (DMSO-*d*_*6*_, 100.62 MHz): δ (ppm) 169.59 (C-N), 152.98 (C-S), 37.11(CH_2_-S), 23.79 (CH_2_), 15.42 (CH_2_-Si), −1.69 (CH_3_-Si). MALDI-MS m/z: [M]^+^ theoretical for C_8_H_18_AuCl_4_N_3_S_2_Si 584.913; found, 584.924. Anal. Calcd (%) for C_8_H_18_AuCl_4_N_3_S_2_Si (Mw 587.24 g mol^−1^): C, 16.36; H, 3.09; N, 7.16; S, 10.92. Found: C, 16.14; H, 3.16; N, 7.64; S, 11.02.

#### (H_3_L^2^)[AuCl_4_] synthesis

116 mg (0.28 mmol) of HAuCl_4_ and 47.2 mg (0.21 mmol) of H_2_L^2^ were dissolved in 10 mL of CH_2_Cl_2_ under Schlenk conditions (N_2_) and mixed at room temperature overnight. Afterwards, 30 mL of n-hexane were added, yielding an orange oil, which was collected by filtration and washed with fresh n-hexane. The orange oil, initially trapped in the filter pores, was subsequently recovered in pure form using CH_2_Cl_2_. Yield: 97.6 mg (80.7%). IR (KBr), cm^−1^: ν_max_ 3389, 3300, 3202, 1624 (-NH_3_), 1570 (thiadiazole), 1460 (-CH_3_). ^1^H NMR (DMSO-*d*_*6*_, 400.13 MHz): δ (ppm) 7.8 (s, 1H, -NH_3_), 3.07 (t, *J* = *7.23 Hz*, 2H, S-CH_2_), 1.64 (quintet, J = 7.13 Hz, 2H, -CH_2_-), 1.37 (quintet, J = 7.14 Hz, 2H, -CH_2_-), 1.30–1.26 (m, 4H, -CH_2_-), 0.87 (t, J = *7.08 Hz,* 3H, CH_3_). ^13^C NMR (DMSO-*d*_*6*_, 100.62 MHz): δ (ppm) 169.97 (C-N), 151.53 (C-S), 37.81 (CH_2_-S), 34.64 (CH_2_), 31.12 (CH_2_), 29.41 (CH_2_), 27.98 (CH_2_), 22.42 (CH_2_), 14.33 (CH_3_). MALDI-MS m/z: [M]^+^ theoretical for C_8_H_16_AuCl_4_N_3_S_2_ 554.920; found, 554.930.

#### [(HL^1^)_2_Au_2_Cl_2_] synthesis

98.3 mg (0.45 mmol) of H_2_L^1^ and 153.8 mg (0.45 mmol) of HAuCl_4_ were dissolved in 10 mL of methylene chloride under Schlenk conditions (N_2_) and stirred for 1 h. Then, 3 mL of water were added, and the reaction mixture was stirred for another 12 h at room temperature. After completion, three successive extractions with water were performed, and the organic phase was collected and precipitated with n-hexane. The resulting purple precipitate was purified by repeated reprecipitations from dichloromethane with n-hexane, followed by centrifugation. Crystals were grown from DMSO as red needles. Yield: 36.5 mg (19.2%). IR (KBr), cm^−1^: ν_max_ 3277 (-NH), 1614 (-NH), 1535 (thiadiazole), 1246, 862, 837 (Si-CH_3_). ^1^H NMR (DMSO-*d*_*6*_, 400.13 MHz): δ (ppm) 8.24 (s, 1H, -NH), 3.07 (t, *J* = *7.39 Hz*, 2H, S-CH_2_), 1.71 − 1.63 (m, 2H, -CH_2_-), 0.60 (quintet, J = 3.77 Hz, 2H, Si-CH_2_), −0.002 (s, 9H, Si-CH_3_). Anal. Calcd (%) for C_10_H_22_AuClN_3_OS_3_Si (Mw 556.99 g mol^−1^): C12.01; H 1.01; N 14.01; S32.06. Found: C, 12.23; H, 1.20; N, 13.9; S, 31.5.

#### [(HL^2^)_2_Au_2_Cl_2_] synthesis

108 mg (0.43 mmol) of H_2_L^3^ and 152.3 mg (0.43 mmol) of HAuCl_4_ were dissolved in 10 mL of methylene chloride under Schlenk conditions (N_2_) and stirred for 1 h. Then, 3 mL of water were added, and the reaction mixture was stirred for another 12 h at room temperature. After completion, three successive extractions with water were performed, and the organic phase was collected and precipitated with n-hexane. The resulting purple precipitate was purified by repeated reprecipitations from dichloromethane with n-hexane, followed by centrifugation. Crystals were grown from DMSO as red needles. Yield: 73.86 mg (33.1%). IR (KBr), cm^−1^: ν_max_ 3296 (-NH), 1622 (-NH), 1539 (thiadiazole). ^1^H NMR (DMSO-*d*_*6*_, 400.13 MHz): δ (ppm) 8.25 (s, NH, 1H), 3.08 (t, *J* = *7.38 Hz*, 2H, S-CH_2_), 1.66 (quintet, J = 7.15 Hz, 2H, -CH_2_-), 1.38 − 1.25 (m, 6H, -CH_2_-), 0.87 (t, *J* = *7.21 Hz,* 3H, CH_3_). MALDI-MS m/z: [M—2Cl]^+^ theoretical for C_16_H_28_Au_2_N_6_S_4_ 828.075; found, 828.079. Anal. Calcd (%) for C_16_H_28_Au_2_Cl_2_N_6_S_4_ (Mw 897.53 g mol^−1^): C, 21.41; H, 3.14; N, 9.36; S, 14.29. Found: C, 21.24; H, 2.93; N, 9.08; S, 14.16.

## Supplementary Information

Supplementary Information.

## Data Availability

The data that support the findings of this study are available in the supplementary material of this article.
