# Supplementary material for: Au complexes with 5-S-alkylated-2-amino-1,3,4-thiadiazoles: from Au(III)-based ionic liquids to rare dinuclear Au(II) coordination compounds
Source: Sci Rep. 2026 Jun 18;16:27998. doi: 10.1038/s41598-026-57927-y (PMC13549981; doi:10.1038/s41598-026-57927-y)
Supplement: Supplementary file 1 [file 41598_2026_57927_MOESM1_ESM.docx]

**Au complexes with 5-S-alkylated-2-amino-1,3,4-thiadiazoles: from Au(III)-based ionic liquids to rare dinuclear Au(II) coordination compounds**

Madalin Damoc,^*[a]^ Alexandru-Constantin Stoica,^[a]^ Mihaela Silion,^[b]^ Ana-Maria Macsim,^[c]^ Aurica Farcas,^[d]^ Mihaela Dascalu,^[a]^ Sergiu Shova,^[a]^ Maria Cazacu^*[a]^

*^[a]^Department of Inorganic Polymers, “Petru Poni” Institute of Macromolecular Chemistry, Romanian Academy, Aleea Gr. Ghica Voda 41A, 700487, Iasi, Romania*

*^[b]^Department of Physics of Polymers and Polymeric Materials, ““Petru Poni” Institute of Macromolecular Chemistry, Romanian Academy, Aleea Gr. Ghica Voda 41A, 700487, Iasi, Romania*

*^[c]^NMR Laboratory, ““Petru Poni” Institute of Macromolecular Chemistry, Romanian Academy, Aleea Gr. Ghica Voda 41A, 700487, Iasi, Romania*

*^[d]^Department of Electroactive Polymers and Plasmochemistry, ““Petru Poni” Institute of Macromolecular Chemistry, Romanian Academy, Aleea Gr. Ghica Voda 41A, 700487, Iasi, Romania*

*Corresponding authors, e-mail: [damoc.madalin@icmpp.ro](mailto:damoc.madalin@icmpp.ro); mcazacu@icmpp.ro

# **Characterisation data of H_2_L^2^**

**Table S1.** Crystal data and structure refinement for **H_2_L^2^**.

| **Compound** | **H_2_L^3^** |
| --- | --- |
| Emp. Form. | C_8_H_15_N_3_S_2_ |
| Fw | 217.35 |
| T[K] | 293 |
| Space group | *P2*_1_/*c* |
| a[Å] | 14.203(3) |
| b[Å] | 8.1726(9) |
| c[Å] | 10.7871(12) |
| *α*[°] = *γ*[°] | 90 |
| *β*[°] | 107.440(15) |
| *V*[Å^3^] | 1194.6(3) |
| *Z* | 4 |
| ρ_calc_[g/cm^3^] | 1.209 |
| μ[mm^‑1^] | 0.410 |
| Crystal size [mm^3^] | 0.4 × 0.12 × 0.02 |
| Radiation | Mo Kα (λ = 0.71073) |
| 2Θ range [°] | 5.822 to 50.044 |
| Reflections collected | 6939 |
| Indep. refl., *R*_int_ | 2105, 0.0480 |
| Data/restraints/par. | 2105/58/138 |
| GOF | 1.027 |
| *R*_1_, *wR*_2_ for [I>=2σ (I)] | 0.0860, 0.2073 |
| *R*_1_, *wR*_2_ (all data) | 0.1912, 0.2736 |
| Δ*ρ*_max_, Δ*ρ*_min_ (e Å^-3^) | 0.24/-0.17 |
| CCDC no. | 2257485 |

**Table S2.** Bond lengths [Å], bond angles [°] and hydrogen bond parameters for **H_2_L^2^**.

| S1–C1 | 1.729(7) | N3–C2 | 1.274(8) |
| --- | --- | --- | --- |
| S1–C2 | 1.733(6) | C3–C4 | 1.390(10) |
| S2–C2 | 1.724(7) | C4–C5 | 1.385(10) |
| S2–C3 | 1.732(12) | C5–C6 | 1.391(10) |
| N1–C1 | 1.340(7) | C6–C7 | 1.385(10) |
| N2–N3 | 1.387(7) | C7–C8 | 1.387(10) |
| N2–C1 | 1.310(6) |  |  |

| C1–S1–C2 | 86.8(3) | N3–C2–S1 | 114.2(5) |
| --- | --- | --- | --- |
| C2–S2–C3 | 95.1(18) | N3–C2–S2 | 120.9(5) |
| C1–N2–N3 | 111.7(5) | C4–C3–S2 | 96(3) |
| C2–N3–N2 | 113.5(5) | C5–C4–C3 | 160(6) |
| N1–C1–S1 | 123.2(4) | C4–C5–C6 | 149(3) |
| N2–C1–S1 | 113.8(5) | C7–C6–C5 | 141(4) |
| N2–C1–N1 | 122.9(6) | C6–C7–C8 | 120(5) |
| S2–C2–S1 | 124.8(4) |  |  |

| **D**–**H···A** | **d(D-H)/Å** | **d(H-A)/Å** | **d(D-A)/Å** | **D-H-A/^o^** | **Symmetry element** |
| --- | --- | --- | --- | --- | --- |
| N1–H···N3 | 0.86 | 2.10 | 2.938(7) | 164.9 | +x, 3/2 – y, ½ + z |
| C3–H···S1 | 0.97 | 2.36 | 2.95(7) | 118.7 | - |

**Figure S1.** IR spectrum for **H_2_L^2^**.

**Figure S2.** ^1^H NMR spectrum for **H_2_L^2^** (DMSO-d_6_, 400.13 MHz).

**Figure S3.** ^13^C NMR spectrum for **H_2_L^2^** (DMSO-d_6_, 100.6 MHz).

# **Characterisation data of ionic AuCl_4_^-^ compounds**

**Table S3.** Crystal data and structure refinement for the **(H_3_L^1^)[AuCl_4_].**

| **Compound** | **(H_3_L^1^)[AuCl_4_]** |
| --- | --- |
| Emp. Form. | C_8_H_18_AuCl_4_N_3_S_2_Si |
| Fw | 587.23 |
| T[K] | 200 |
| Space group | *Pca2*_1_ |
| a[Å] | 47.31(10) |
| b[Å] | 10.376(5) |
| c[Å] | 7.83(2) |
| *α = β = γ* [°] | 90 |
| *V*[Å^3^] | 3844(14) |
| *Z* | 8 |
| ρ_calc_[g/cm^3^] | 2.029 |
| μ[mm^‑1^] | 22.062 |
| Crystal size [mm^3^] | 0.08 × 0.031 × 0.025 |
| 2Θ range [°] | 7.474 to 148.892 |
| Reflections collected | 16830 |
| Indep. refl., *R*_int_ | 5594, 0.0812 |
| Data/restraints/par. | 5594/83/353 |
| GOF | 1.043 |
| *R*_1_, *wR*_2_ for [I>=2σ (I)] | 0.0777, 0.1881 |
| *R*_1_, *wR*_2_ (all data) | 0.0952, 0.1996 |
| Δ*ρ*_max_, Δ*ρ*_min_ (e Å^-3^) | 2.23/-2.35 |
| Flack parameter | 0.40(4) |
| CCDC no. | 2390300 |

**Table S4.** Bond lengths [Å], bond angles [°] and hydrogen bond parameters for **(H_3_L^1^)[AuCl_4_]**

| Au1A-Cl1A | 2.239(8) | N2A-C1A | 1.29(4) |
| --- | --- | --- | --- |
| Au1A-Cl2A | 2.250(8) | N3A-C2A | 1.28(2) |
| Au1A-Cl3A | 2.224(8) | C3A-C4A | 1.548(6) |
| Au1A-Cl4A | 2.253(9) | C4A-C5A | 1.548(6) |
| Au1B-Cl1B | 2.216(9) | S1B-C1B | 1.75(3) |
| Au1B-Cl2B | 2.236(9) | S1B-C2B | 1.78(3) |
| Au1B-Cl3B | 2.243(9) | S2B-C2B | 1.74(2) |
| Au1B-Cl4B | 2.261(8) | S2B-C3B | 1.78(2) |
| S1A-C1A | 1.69(4) | Si1B-C5B | 1.92(4) |
| S1A-C2A | 1.69(2) | Si1B-C6B | 1.85(3) |
| S2A-C2A | 1.74(2) | Si1B-C7B | 1.85(3) |
| S2A-C3A | 1.78(2) | Si1B-C8B | 1.85(3) |
| Si1A-C5A | 1.81(3) | N1B-C1B | 1.41(4) |
| Si1A-C6A | 1.95(3) | N2B-N3B | 1.34(3) |
| Si1A-C7A | 1.95(3) | N2B-C1B | 1.28(2) |
| Si1A-C8A | 1.95(3) | N3B-C2B | 1.28(2) |
| N1A-C1A | 1.32(4) | C3B-C4B | 1.548(7) |
| N2A-N3A | 1.34(3) | C4B-C5B | 1.548(6) |

| Cl1A-Au1A-Cl2A | 91.1(4) | N3A-C2A-S1A | 115.9(18) |
| --- | --- | --- | --- |
| Cl1A-Au1A-Cl4A | 89.6(4) | N3A-C2A-S2A | 122.1(19) |
| Cl2A-Au1A-Cl4A | 177.0(4) | C4A-C3A-S2A | 106.0(13) |
| Cl3A-Au1A-Cl1A | 177.6(5) | C5A-C4A-C3A | 106.5(19) |
| Cl3A-Au1A-Cl2A | 88.2(4) | C4A-C5A-Si1A | 109.4(19) |
| Cl3A-Au1A-Cl4A | 91.3(4) | C1B-S1B-C2B | 86.5(14) |
| Cl1B-Au1B-Cl2B | 90.1(4) | C2B-S2B-C3B | 97.0(12) |
| Cl1B-Au1B-Cl3B | 177.7(5) | C6B-Si1B-C5B | 114(4) |
| Cl1B-Au1B-Cl4B | 89.6(3) | C7B-Si1B-C5B | 108(4) |
| Cl2B-Au1B-Cl3B | 89.2(4) | C7B-Si1B-C6B | 115(6) |
| Cl2B-Au1B-Cl4B | 178.3(4) | C7B-Si1B-C8B | 114(5) |
| Cl3B-Au1B-Cl4B | 91.2(4) | C8B-Si1B-C5B | 106(4) |
| C2A-S1A-C1A | 87.1(14) | C8B-Si1B-C6B | 99(4) |
| C2A-S2A-C3A | 101.0(11) | C1B-N2B-N3B | 118(3) |
| C5A-Si1A-C6A | 103(3) | C2B-N3B-N2B | 111(2) |
| C5A-Si1A-C8A | 115(3) | N1B-C1B-S1B | 122(2) |
| C7A-Si1A-C6A | 112(4) | N2B-C1B-S1B | 110(2) |
| C7A-Si1A-C8A | 113(3) | N2B-C1B-N1B | 128(3) |
| C8A-Si1A-C6A | 106(4) | S2B-C2B-S1B | 119.6(14) |
| C1A-N2A-N3A | 116(3) | N3B-C2B-S1B | 113.2(19) |
| C2A-N3A-N2A | 109(2) | N3B-C2B-S2B | 127(2) |
| N1A-C1A-S1A | 125(3) | C4B-C3B-S2B | 108.1(18) |
| N2A-C1A-S1A | 112(2) | C5B-C4B-C3B | 115(3) |
| N2A-C1A-N1A | 124(4) | C4B-C5B-Si1B | 113(3) |
| S1A-C2A-S2A | 122.0(13) |  |  |

| **D-H···A** | **d(D-H)/Å** | **d(H-A)/Å** | **d(D-A)/Å** | **D-H-A/°** | **Symmetry element** |
| --- | --- | --- | --- | --- | --- |
| N1A-H···S1A | 0.91 | 2.66 | 3.55(3) | 165.2 | 3/2 – *x*, +*y*, –½ + *z* |
| N1A-H···Cl1A | 0.91 | 2.63 | 3.46(3) | 152.6 | - |
| N1A-H···Cl4B | 0.91 | 2.65 | 3.37(3) | 136.3 | 3/2 – *x*, +*y*, ½ + *z* |
| N1B-H···S2B | 0.91 | 3.05 | 3.83(3) | 145.3 | +*x*, +*y*, –1 + *z* |
| N1B-H···Cl2B | 0.91 | 2.66 | 3.32(3) | 130.9 | - |
| N1B-H···Cl3B | 0.91 | 2.79 | 3.50(3) | 134.8 | 3/2 – *x*, +*y*, ½ + *z* |
| N1B-H···Cl2A | 0.91 | 2.61 | 3.43(3) | 150.6 | - |
| N1B-H···Cl4B | 0.91 | 2.80 | 3.39(3) | 123.6 | 3/2 – *x*, +*y*, ½ + *z* |

**Figure S4.** IR spectrum for **(H_3_L^1^)[AuCl_4_].**

**Figure S5.** IR spectrum for **(H_3_L^2^)[AuCl_4_].**

**Figure S6.** ^1^H NMR spectrum for **(H_3_L^1^)[AuCl_4_]** (DMSO-d_6_, 400.13 MHz).

**Figure S7.** ^13^C NMR spectrum for **(H_3_L^1^)[AuCl_4_]** (DMSO-d_6_, 100.6 MHz).

**Figure S8.** ^1^H NMR spectrum for **(H_3_L^2^)[AuCl_4_]** (DMSO-d_6_, 400.13 MHz).

**Figure S9.** ^13^C NMR spectrum for **(H_3_L^2^)[AuCl_4_]** (DMSO-d_6_, 100.6 MHz).

# **Ionic-liquid-like behaviour**

**Figure S10**. TGA curves of **H_2_L^1^** and **H_2_L^2^**.

# **Characterization data of dinuclear Au(II) complexes**

**Table S5.** Crystal data and structure refinement for dinuclear Au(II) complexes.

| **Compound** | **[(HL^1^)_2_(Au_2_Cl_2_)]** | **[(HL^2^)_2_(Au_2_Cl_2_)]** |
| --- | --- | --- |
| Emp. Form. | C_10_H_22_AuClN_3_OS_3_Si | C_16_H_28_Au_2_Cl_2_N_6_S_4_ |
| Fw | 556.99 | 891.47 |
| T[K] | 200 | 293 |
| Space group | *P*-1 | *P*-1 |
| a[Å] | 6.1866(3) | 5.2683(10) |
| b[Å] | 12.2175(7) | 8.1043(17) |
| c[Å] | 13.2824(6) | 16.573(2) |
| *α*[°] | 96.887(4) | 97.606(14) |
| *β*[°] | 99.941(4) | 98.123(14) |
| *γ*[°] | 96.707(4) | 104.384(18) |
| *V*[Å^3^] | 971.99(9) | 668.1(2) |
| *Z* | 2 | 1 |
| ρ_calc_[g/cm^3^] | 1.903 | 2.216 |
| μ[mm^‑1^] | 8.087 | 11.495 |
| Crystal size [mm^3^] | 0.15 × 0.12 × 0.03 | 0.40 × 0.04 × 0.01 |
| Radiation Mo Kα (λ =0.71073) | | |
| 2Θ range [°] | 4.284 to 50.044 | 5.044 to 50.048 |
| Reflections collected | 6502 | 3732 |
| Indep. refl., *R*_int_ | 3437, 0.0435 | 3732, 0.092 |
| Data/restraints/par. | 3437/0/196 | 3732/54/119 |
| GOF | 1.018 | 0.936 |
| *R*_1_, *wR*_2_ for [I>=2σ (I)] | 0.0376, 0.0605 | 0.0874, 0.1984 |
| *R*_1_, *wR*_2_ (all data) | 0.0464, 0.0650 | 0.1228, 0.2088 |
| Δ*ρ*_max_, Δ*ρ*_min_ (e Å^-3^) | 1.04/-1.16 | 3.38/-2.10 |
| CCDC no. | 2257489 | 2257490 |

**Table S6.** Bond lengths [Å], bond angles [°], and hydrogen bond parameters for **[(HL^1^)_2_(Au_2_Cl_2_)]**.

| Au1–Au1^1^ | 2.5383(5) | Si1–C8 | 1.852(7) |
| --- | --- | --- | --- |
| Au1–Cl1 | 2.3663(17) | N1–C1 | 1.316(8) |
| Au1–N1 | 1.972(5) | N2–Au1^1^ | 2.019(5) |
| Au1–N2^1^ | 2.019(5) | N2–N3 | 1.394(6) |
| S1–C1 | 1.751(6) | N2–C1 | 1.325(8) |
| S1–C2 | 1.734(7) | N3–C2 | 1.306(8) |
| S2–C2 | 1.750(6) | C3–C4 | 1.523(8) |
| S2–C3 | 1.810(7) | C4–C5 | 1.526(9) |
| Si1–C5 | 1.871(7) | S3–O1 | 1.57(2) |
| Si1–C6 | 1.873(7) | S3–C9 | 1.770(8) |
| Si1–C7 | 1.863(7) | S3–C10 | 1.662(9) |

^1^ 2 – *x*, 1 – *y*, 2 – *z*

| Cl1–Au1–Au1^1^ | 176.50(5) | C1–N2–Au1^1^ | 120.0(4) |
| --- | --- | --- | --- |
| N1–Au1–Au1^1^ | 87.61(16) | C1–N2–N3 | 116.8(5) |
| N1–Au1–Cl1 | 89.09(17) | C2–N3–N2 | 108.2(6) |
| N1–Au1–N2^1^ | 174.1(2) | N1–C1–S1 | 125.1(5) |
| N2^1^–Au1–Au1^1^ | 86.50(14) | N1–C1–N2 | 124.1(6) |
| N2^1^–Au1–Cl1 | 96.78(15) | N2–C1–S1 | 110.7(5) |
| C2–S1–C1 | 87.0(3) | S1–C2–S2 | 119.4(4) |
| C2–S2–C3 | 99.7(3) | N3–C2–S1 | 117.2(5) |
| C5–Si1–C6 | 109.3(4) | N3–C2–S2 | 123.3(5) |
| C7–Si1–C5 | 110.5(4) | C4–C3–S2 | 109.3(5) |
| C7–Si1–C6 | 110.3(3) | C3–C4–C5 | 113.5(5) |
| C8–Si1–C5 | 109.0(3) | C4–C5–Si1 | 114.5(4) |
| C8–Si1–C6 | 110.0(4) | O1–S3–C9 | 100.5(8) |
| C8–Si1–C7 | 107.7(4) | O1–S3–C10 | 112.8(8) |
| C1–N1–Au1 | 121.2(5) | C10–S3–C9 | 101.4(4) |
| N3–N2–Au1^1^ | 122.7(4) | ^1^ 2 – *x*, 1 – *y*, 2 – *z* | |

| **D-H···A** | **d(D-H)/Å** | **d(H-A)/Å** | **d(D-A)/Å** | **D-H-A/°** | **Symmetry element** |
| --- | --- | --- | --- | --- | --- |
| N1-H···O1 | 0.79(6) | 2.07(7) | 2.82(2) | 156(6) | - |
| C9-H···Cl1 | 0.98 | 2.89 | 3.717(8) | 142.5 | 3 – *x*, 2 – *y*,2 – *z* |
| C10-H···Cl1 | 0.98 | 2.80 | 3.682(10) | 150.0 | 3 – *x*, 2 – *y*,2 – *z* |
| C10-H···S1 | 0.98 | 3.02 | 3.903(9) | 150.4 | 1 + *x*, +*y*, +*z* |

**Table S7.** Bond lengths [Å], bond angles [°], and hydrogen bond parameters for **[(HL^2^)_2_(Au_2_Cl_2_)]**.

| Au1-Au1^1^ | 2.5299(18) | N2-N3 | 1.38(2) |
| --- | --- | --- | --- |
| Au1-Cl1 | 2.361(6) | N2-C1 | 1.35(3) |
| Au1-N1^1^ | 2.01(2) | N3-C2 | 1.26(3) |
| Au1-N2 | 1.98(2) | C3-C4 | 1.529(13) |
| S1-C1 | 1.74(3) | C4-C5 | 1.529(13) |
| S1-C2 | 1.74(3) | C5-C6 | 1.529(13) |
| S2-C2 | 1.78(2) | C6-C7 | 1.529(13) |
| S2-C3 | 1.82(3) | C7-C8 | 1.529(13) |
| N1-C1 | 1.32(3) |  |  |

^1^ –*x*, 1 – *y*, 1 – *z*

| Cl1-Au1-Au1^1^ | 177.0(2) | C2-N3-N2 | 110(2) |
| --- | --- | --- | --- |
| N1^1^-Au1-Au1^1^ | 88.2(5) | N1-C1-S1 | 123(2) |
| N1^1^-Au1-Cl1 | 89.5(6) | N1-C1-N2 | 125(2) |
| N2-Au1-Au1^1^ | 86.9(5) | N2-C1-S1 | 111.7(17) |
| N2-Au1-Cl1 | 95.4(6) | S1-C2-S2 | 118.2(17) |
| N2-Au1-N1^1^ | 175.1(7) | N3-C2-S1 | 118.1(19) |
| C1-S1-C2 | 86.0(12) | N3-C2-S2 | 124(2) |
| C2-S2-C3 | 100(2) | C4-C3-S2 | 120(2) |
| C1-N1-Au1^1^ | 119.1(17) | C5-C4-C3 | 121(3) |
| N3-N2-Au1 | 125.0(17) | C6-C5-C4 | 122(3) |
| C1-N2-Au1 | 120.9(16) | C5-C6-C7 | 123(3) |
| C1-N2-N3 | 114(2) | C8-C7-C6 | 95(2) |

^1^ –*x*, 1 – *y*, 1 – *z*

| **D-H···A** | **d(D-H)/Å** | **d(H-A)/Å** | **d(D-A)/Å** | **D-H-A/°** | **Symmetry element** |
| --- | --- | --- | --- | --- | --- |
| N1-H···Cl1 | 0.86 | 2.49 | 3.26(2) | 148.4 | +*x*, 1 + *y*, +*z* |

**Figure S11.** IR spectrum for **[(HL^1^)_2_(Au_2_Cl_2_)]**.

**Figure S12.** IR spectrum for **[(HL^2^)_2_(Au_2_Cl_2_)]**.

**Figure S13.** ^1^H NMR spectrum for **[(HL^1^)_2_(Au_2_Cl_2_)]** (DMSO-d_6_, 400.13 MHz).

**Figure S14.** ^1^H NMR spectrum for **[(HL^2^)_2_(Au_2_Cl_2_)]** (DMSO-d_6_, 400.3 MHz).

**Table S8.** XYZ matrix for the optimized **[(HL^1^)_2_(Au_2_Cl_2_)]** molecule

| Au | 1.026474 | -0.80702 | 0.000069 | Au | -1.02647 | 0.807024 | -0.000037 |
| --- | --- | --- | --- | --- | --- | --- | --- |
| Cl | 2.78253 | -2.46382 | 0.000017 | Cl | -2.78253 | 2.463814 | -0.00045 |
| S | -2.86782 | -3.35722 | -0.00002 | S | 2.867814 | 3.357217 | 0.000085 |
| S | -5.78302 | -2.40661 | 0.000031 | S | 5.783023 | 2.406613 | 0.000011 |
| Si | -10.5228 | 0.731441 | 0.000064 | Si | 10.52278 | -0.73144 | -0.000047 |
| N | -0.32324 | -2.31113 | -0.0002 | N | 0.323241 | 2.31113 | 0.000261 |
| H | 0.039758 | -3.25932 | -0.000092 | H | -0.03976 | 3.259319 | 0.000162 |
| N | -2.19136 | -0.88455 | -0.00019 | N | 2.191355 | 0.884551 | 0.000235 |
| N | -3.55159 | -0.85902 | -0.00013 | N | 3.551594 | 0.859022 | 0.00017 |
| C | -1.62622 | -2.10446 | -0.00012 | C | 1.626219 | 2.104462 | 0.000187 |
| C | -4.07029 | -2.03707 | -0.000037 | C | 4.070289 | 2.037066 | 0.000082 |
| C | -6.46433 | -0.69388 | -0.000063 | C | 6.464326 | 0.693878 | 0.000075 |
| H | -6.07176 | -0.17418 | 0.885984 | H | 6.071771 | 0.174198 | -0.88599 |
| H | -6.07188 | -0.17432 | -0.88625 | H | 6.071867 | 0.174303 | 0.886242 |
| C | -7.99273 | -0.74959 | 0.000037 | C | 7.992728 | 0.749593 | -0.00001 |
| H | -8.33537 | -1.31685 | 0.883076 | H | 8.335372 | 1.316866 | -0.88303 |
| H | -8.33548 | -1.31698 | -0.88287 | H | 8.335467 | 1.316965 | 0.882914 |
| C | -8.61731 | 0.655169 | -0.000031 | C | 8.617306 | -0.65517 | 0.000033 |
| H | -8.25794 | 1.220694 | -0.87985 | H | 8.257936 | -1.22071 | 0.879839 |
| H | -8.25785 | 1.220818 | 0.879672 | H | 8.257858 | -1.2208 | -0.87969 |
| C | -11.0387 | 2.551301 | 0.000139 | C | 11.0387 | -2.5513 | -0.00016 |
| H | -10.6552 | 3.077505 | -0.88985 | H | 10.65516 | -3.07752 | 0.889822 |
| H | -10.6553 | 3.077402 | 0.890232 | H | 10.65527 | -3.07738 | -0.89026 |
| H | -12.1369 | 2.653008 | 0.000085 | H | 12.13687 | -2.65301 | -0.000095 |
| C | -11.2027 | -0.12299 | -1.54708 | C | 11.20268 | 0.122963 | 1.547121 |
| H | -10.9371 | -1.19276 | -1.57505 | H | 10.93712 | 1.192733 | 1.575104 |
| H | -10.8123 | 0.34357 | -2.46704 | H | 10.81229 | -0.34362 | 2.467067 |
| H | -12.303 | -0.05572 | -1.5816 | H | 12.30302 | 0.05569 | 1.581648 |
| C | -11.2025 | -0.12308 | 1.547227 | C | 11.20256 | 0.123111 | -1.54719 |
| H | -12.3029 | -0.0556 | 1.581974 | H | 12.30288 | 0.055631 | -1.58192 |
| H | -10.8119 | 0.343278 | 2.467173 | H | 10.81191 | -0.34323 | -2.46715 |
| H | -10.9372 | -1.19291 | 1.574987 | H | 10.9372 | 1.192938 | -1.57493 |

**Table S9.** XYZ matrix for the optimized **[(HL^2^)_2_(Au_2_Cl_2_)]** molecule

| Au | 1.140435 | 0.559925 | 0.299024 | Au | -1.14042 | -0.56004 | -0.29879 |
| --- | --- | --- | --- | --- | --- | --- | --- |
| Cl | 3.169906 | 1.768649 | 0.827432 | Cl | -3.16983 | -1.76872 | -0.82756 |
| S | -2.18517 | 3.816601 | 0.396355 | S | 2.185271 | -3.81659 | -0.39672 |
| S | -5.18819 | 3.487841 | -0.19064 | S | 5.188342 | -3.48778 | 0.18999 |
| N | -1.94781 | 1.316736 | -0.11496 | N | 1.947832 | -1.31685 | 0.115151 |
| N | -3.27215 | 1.568964 | -0.28042 | N | 3.272204 | -1.56903 | 0.280393 |
| N | 0.114923 | 2.298689 | 0.430977 | N | -0.11493 | -2.29883 | -0.43065 |
| H | 0.632707 | 3.130473 | 0.696885 | H | -0.63267 | -3.13055 | -0.69682 |
| C | -1.18909 | 2.368257 | 0.239658 | C | 1.189128 | -2.36834 | -0.23958 |
| C | -3.57973 | 2.798617 | -0.05984 | C | 3.579831 | -2.79863 | 0.059536 |
| C | -6.10248 | 2.006055 | -0.7958 | C | 6.102637 | -2.00607 | 0.795309 |
| H | -7.02046 | 2.436359 | -1.22697 | H | 7.020708 | -2.43642 | 1.22624 |
| H | -5.5062 | 1.581059 | -1.61608 | H | 5.506474 | -1.58128 | 1.615778 |
| C | -6.41123 | 0.953131 | 0.266773 | C | 6.411162 | -0.9529 | -0.26708 |
| H | -7.06984 | 1.388339 | 1.038374 | H | 7.069615 | -1.38793 | -1.03892 |
| H | -5.47473 | 0.660366 | 0.764277 | H | 5.474551 | -0.66003 | -0.76432 |
| C | -7.05448 | -0.29795 | -0.34372 | C | 7.054532 | 0.298044 | 0.343551 |
| H | -7.99546 | -0.0248 | -0.85783 | H | 7.995688 | 0.024805 | 0.857298 |
| H | -6.37797 | -0.69969 | -1.11874 | H | 6.378243 | 0.699544 | 1.1189 |
| C | -7.98051 | -2.64953 | 0.090629 | C | 7.98037 | 2.64977 | -0.09046 |
| H | -8.92844 | -2.36928 | -0.40472 | H | 8.928487 | 2.369402 | 0.404462 |
| H | -7.32872 | -3.04561 | -0.70883 | H | 7.328857 | 3.045599 | 0.709351 |
| C | -7.33189 | -1.39641 | 0.686656 | C | 7.331556 | 1.396806 | -0.68661 |
| H | -7.97869 | -0.9963 | 1.490256 | H | 7.978067 | 0.996932 | -1.49056 |
| H | -6.37884 | -1.67712 | 1.170643 | H | 6.378316 | 1.677632 | -1.17016 |
| C | -8.24838 | -3.74702 | 1.121507 | C | 8.247828 | 3.747559 | -1.12112 |
| H | -8.92621 | -3.39413 | 1.917236 | H | 8.925365 | 3.394917 | -1.9172 |
| H | -7.31436 | -4.07552 | 1.607434 | H | 7.313617 | 4.076179 | -1.60661 |
| H | -8.7121 | -4.63329 | 0.659283 | H | 8.711695 | 4.63371 | -0.65881 |

**Table S10.** The calculated Wiberg order for **[(HL^1^)_2_(Au_2_Cl_2_)]**

| Bond | Order | Bond | Order |
| --- | --- | --- | --- |
| 1(Au)-2(Cl) | 1.029306 | 21(C)-24(H) | 0.968222 |
| 1(Au)-6(N) | 0.744526 | 25(C)-26(H) | 0.969026 |
| 1(Au)-7(H) | 0.055913 | 25(C)-27(H) | 0.968971 |
| 1(Au)-8(N) | 0.066948 | 25(C)-28(H) | 0.96786 |
| 1(Au)-10(C) | 0.087583 | 29(C)-30(H) | 0.96786 |
| 1(Au)-33(Au) | 0.689796 | 29(C)-31(H) | 0.968972 |
| 1(Au)-34(Cl) | 0.160615 | 29(C)-32(H) | 0.969027 |
| 1(Au)-38(N) | 0.070932 | 33(Au)-34(Cl) | 1.029306 |
| 1(Au)-40(N) | 0.674137 | 33(Au)-38(N) | 0.744526 |
| 1(Au)-41(N) | 0.087386 | 33(Au)-39(H) | 0.055913 |
| 1(Au)-42(C) | 0.083165 | 33(Au)-40(N) | 0.066948 |
| 2(Cl)-33(Au) | 0.160615 | 33(Au)-42(C) | 0.087583 |
| 2(Cl)-34(Cl) | 0.098935 | 35(S)-36(S) | 0.060242 |
| 3(S)-4(S) | 0.060242 | 35(S)-38(N) | 0.106614 |
| 3(S)-6(N) | 0.106614 | 35(S)-40(N) | 0.093086 |
| 3(S)-8(N) | 0.093086 | 35(S)-41(N) | 0.154171 |
| 3(S)-9(N) | 0.154171 | 35(S)-42(C) | 1.241915 |
| 3(S)-10(C) | 1.241915 | 35(S)-43(C) | 1.184196 |
| 3(S)-11(C) | 1.184197 | 36(S)-41(N) | 0.15548 |
| 4(S)-9(N) | 0.15548 | 36(S)-43(C) | 1.277388 |
| 4(S)-11(C) | 1.277388 | 36(S)-44(C) | 1.074029 |
| 4(S)-12(C) | 1.074029 | 37(Si)-50(C) | 0.942353 |
| 5(Si)-18(C) | 0.942353 | 37(Si)-53(C) | 0.989281 |
| 5(Si)-21(C) | 0.989281 | 37(Si)-57(C) | 0.988385 |
| 5(Si)-25(C) | 0.988385 | 37(Si)-61(C) | 0.988384 |
| 5(Si)-29(C) | 0.988384 | 38(N)-39(H) | 0.964254 |
| 6(N)-7(H) | 0.964254 | 38(N)-40(N) | 0.161109 |
| 6(N)-8(N) | 0.161109 | 38(N)-42(C) | 1.542938 |
| 6(N)-10(C) | 1.542938 | 39(H)-42(C) | 0.068356 |
| 6(N)-33(Au) | 0.070932 | 40(N)-41(N) | 1.254639 |
| 6(N)-40(N) | 0.085663 | 40(N)-42(C) | 1.440573 |
| 7(H)-10(C) | 0.068357 | 40(N)-43(C) | 0.134301 |
| 8(N)-9(N) | 1.254639 | 41(N)-42(C) | 0.102071 |
| 8(N)-10(C) | 1.440573 | 41(N)-43(C) | 1.86667 |
| 8(N)-11(C) | 0.134301 | 42(C)-43(C) | 0.092448 |
| 8(N)-33(Au) | 0.674137 | 44(C)-45(H) | 0.950383 |
| 8(N)-38(N) | 0.085663 | 44(C)-46(H) | 0.950383 |
| 9(N)-10(C) | 0.102071 | 44(C)-47(C) | 1.057876 |
| 9(N)-11(C) | 1.86667 | 44(C)-50(C) | 0.056158 |
| 9(N)-33(Au) | 0.087386 | 47(C)-48(H) | 0.936602 |
| 10(C)-11(C) | 0.092447 | 47(C)-49(H) | 0.936602 |
| 10(C)-33(Au) | 0.083165 | 47(C)-50(C) | 1.047328 |
| 12(C)-13(H) | 0.950383 | 50(C)-51(H) | 0.942462 |
| 12(C)-14(H) | 0.950383 | 50(C)-52(H) | 0.942462 |
| 12(C)-15(C) | 1.057876 | 53(C)-54(H) | 0.968662 |
| 12(C)-18(C) | 0.056158 | 53(C)-55(H) | 0.968663 |
| 15(C)-16(H) | 0.936602 | 53(C)-56(H) | 0.968222 |
| 15(C)-17(H) | 0.936601 | 57(C)-58(H) | 0.969027 |
| 15(C)-18(C) | 1.047328 | 57(C)-59(H) | 0.968971 |
| 18(C)-19(H) | 0.942462 | 57(C)-60(H) | 0.96786 |
| 18(C)-20(H) | 0.942462 | 61(C)-62(H) | 0.96786 |
| 21(C)-22(H) | 0.968662 | 61(C)-63(H) | 0.968971 |
| 21(C)-23(H) | 0.968663 | 61(C)-64(H) | 0.969027 |

**Table S11.** The calculated Wiberg order for **[(HL^2^)_2_(Au_2_Cl_2_)]**

| Bond | Order | Bond | Order |
| --- | --- | --- | --- |
| 1(Au)-2(Cl) | 1.016607 | 20(C)-26(C) | 1.061348 |
| 1(Au)-5(N) | 0.068415 | 23(C)-24(H) | 0.936491 |
| 1(Au)-7(N) | 0.744241 | 23(C)-25(H) | 0.933573 |
| 1(Au)-8(H) | 0.055199 | 23(C)-26(C) | 0.051338 |
| 1(Au)-9(C) | 0.08779 | 26(C)-27(H) | 0.96281 |
| 1(Au)-30(Au) | 0.692009 | 26(C)-28(H) | 0.962351 |
| 1(Au)-31(Cl) | 0.158734 | 26(C)-29(H) | 0.961303 |
| 1(Au)-34(N) | 0.676331 | 30(Au)-31(Cl) | 1.016612 |
| 1(Au)-35(N) | 0.086803 | 30(Au)-34(N) | 0.068416 |
| 1(Au)-36(N) | 0.070107 | 30(Au)-36(N) | 0.744242 |
| 1(Au)-38(C) | 0.082801 | 30(Au)-37(H) | 0.055199 |
| 2(Cl)-30(Au) | 0.158733 | 30(Au)-38(C) | 0.08779 |
| 2(Cl)-31(Cl) | 0.096687 | 32(S)-33(S) | 0.059668 |
| 3(S)-4(S) | 0.059668 | 32(S)-34(N) | 0.092993 |
| 3(S)-5(N) | 0.092992 | 32(S)-35(N) | 0.152794 |
| 3(S)-6(N) | 0.152794 | 32(S)-36(N) | 0.106365 |
| 3(S)-7(N) | 0.106366 | 32(S)-38(C) | 1.24259 |
| 3(S)-9(C) | 1.242588 | 32(S)-39(C) | 1.186664 |
| 3(S)-10(C) | 1.186665 | 33(S)-35(N) | 0.155017 |
| 4(S)-6(N) | 0.155017 | 33(S)-39(C) | 1.276362 |
| 4(S)-10(C) | 1.27636 | 33(S)-40(C) | 1.087909 |
| 4(S)-11(C) | 1.087909 | 33(S)-43(C) | 0.05539 |
| 4(S)-14(C) | 0.055391 | 34(N)-35(N) | 1.256578 |
| 5(N)-6(N) | 1.256576 | 34(N)-36(N) | 0.160659 |
| 5(N)-7(N) | 0.160662 | 34(N)-38(C) | 1.438929 |
| 5(N)-9(C) | 1.43893 | 34(N)-39(C) | 0.134202 |
| 5(N)-10(C) | 0.134203 | 35(N)-38(C) | 0.102853 |
| 5(N)-30(Au) | 0.67633 | 35(N)-39(C) | 1.860207 |
| 5(N)-36(N) | 0.085982 | 36(N)-37(H) | 0.965316 |
| 6(N)-9(C) | 0.102853 | 36(N)-38(C) | 1.541611 |
| 6(N)-10(C) | 1.860208 | 37(H)-38(C) | 0.068335 |
| 6(N)-30(Au) | 0.086804 | 38(C)-39(C) | 0.092241 |
| 7(N)-8(H) | 0.965317 | 40(C)-41(H) | 0.951455 |
| 7(N)-9(C) | 1.541613 | 40(C)-42(H) | 0.946382 |
| 7(N)-30(Au) | 0.070106 | 40(C)-43(C) | 1.05541 |
| 7(N)-34(N) | 0.085982 | 40(C)-46(C) | 0.05479 |
| 8(H)-9(C) | 0.068335 | 43(C)-44(H) | 0.93792 |
| 9(C)-10(C) | 0.092241 | 43(C)-45(H) | 0.93067 |
| 9(C)-30(Au) | 0.0828 | 43(C)-46(C) | 1.027099 |
| 11(C)-12(H) | 0.951456 | 43(C)-52(C) | 0.052715 |
| 11(C)-13(H) | 0.946381 | 46(C)-47(H) | 0.937282 |
| 11(C)-14(C) | 1.05541 | 46(C)-48(H) | 0.931253 |
| 11(C)-17(C) | 0.05479 | 46(C)-49(C) | 0.052586 |
| 14(C)-15(H) | 0.93792 | 46(C)-52(C) | 1.033522 |
| 14(C)-16(H) | 0.930673 | 49(C)-50(H) | 0.938414 |
| 14(C)-17(C) | 1.0271 | 49(C)-51(H) | 0.937328 |
| 14(C)-23(C) | 0.052715 | 49(C)-52(C) | 1.038416 |
| 17(C)-18(H) | 0.937281 | 49(C)-55(C) | 1.061348 |
| 17(C)-19(H) | 0.931251 | 52(C)-53(H) | 0.936491 |
| 17(C)-20(C) | 0.052586 | 52(C)-54(H) | 0.933571 |
| 17(C)-23(C) | 1.033523 | 52(C)-55(C) | 0.051338 |
| 20(C)-21(H) | 0.938414 | 55(C)-56(H) | 0.96281 |
| 20(C)-22(H) | 0.937328 | 55(C)-57(H) | 0.962351 |
| 20(C)-23(C) | 1.038416 | 55(C)-58(H) | 0.961304 |
